# Test-Time Training Scaling Laws for Chemical Exploration in Drug Design

**DOI:** 10.1021/acs.jcim.5c02316

**Published:** 2025-12-09

**Authors:** Morgan Thomas, Albert Bou, Gianni De Fabritiis

**Affiliations:** † Computational Science Laboratory, 16770 Universitat Pompeu Fabra, Barcelona Biomedical Research Park (PRBB), C Dr. Aiguader 88, 08003 Barcelona, Spain; ‡ Department of Medical Sciences, Khalifa University of Science and Technology, 127788 Abu Dhabi, UAE; § Institució Catalana de Recerca i Estudis Avançats (ICREA), Passeig Lluis Companys 23, 08010 Barcelona, Spain; ∥ Acellera Labs, C Dr Trueta 183, 08005 Barcelona, Spain

## Abstract

Chemical language models (CLMs) leveraging reinforcement learning (RL) have shown promise in de novo molecular design, yet often suffer from mode collapse, limiting their exploration capabilities. Inspired by test-time training (TTT) in large language models, we propose scaling TTT for CLMs to enhance chemical space exploration. We introduce MolExp, a novel benchmark emphasizing the discovery of structurally diverse molecules with similar bioactivity, simulating real-world drug design challenges. Our results demonstrate that scaling TTT by increasing the number of independent RL agents follows a log–linear scaling law, significantly improving exploration efficiency as measured by MolExp. In contrast, increasing TTT training time yields diminishing returns, even with exploration bonuses. We further evaluate cooperative RL strategies to enhance exploration efficiency. These findings provide a scalable framework for generative molecular design, offering insights into optimizing AI-driven drug discovery.

## Graphical abstract

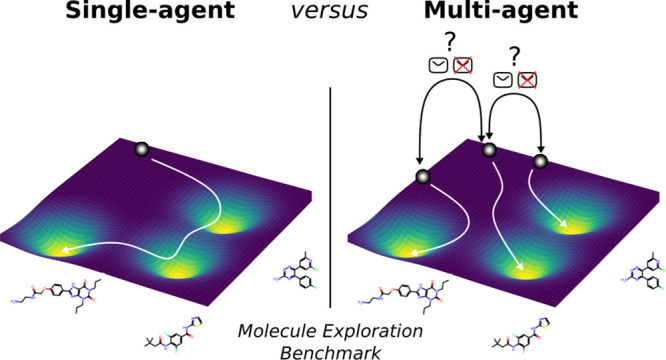

## Introduction

The vast chemical space, estimated to contain over 10^60^ drug-like molecules,^1^ presents a formidable challenge in drug discovery. Efficiently identifying diverse, safe, and efficacious drug candidates can accelerate development timelines and reduce costs by hundreds of millions of dollars.^2^ Traditional virtual screening of limited chemical libraries captures only a fraction of possible compounds, restricting exploration. In contrast, goal-directed generative AI, particularly Chemical Language Models (CLMs), enables de novo design of novel molecules with optimized properties, implicitly navigating expansive chemical spaces.^3^

Thorough exploration of chemical space is critical to maximize the potential of generative AI in drug design. Identifying structurally diverse molecules with similar bioactivity increases the likelihood of discovering backup series with distinct chemical and biological profiles, mitigating risks in drug development.^4^ Moreover, exploring novel chemical regions can circumvent patented compounds.^5^ However, many generative models, including CLMs, are susceptible to mode collapse when fine-tuned and depending on hyperparameters,^6^ particularly those reliant on machine learning models with limited applicability domains due to epistemic uncertainty.^7,8^

CLMs, autoregressive models trained on SMILES strings,^9^ have become the leading approach for generative drug design,^10–12^ excelling in benchmarks like GuacaMol.^13,14^ By integrating reinforcement learning (RL), seminal works^15,16^ have enabled CLMs to generate molecules with tailored properties. Advances in RL algorithms^17–20^ and practical applications^21,22^ have further enhanced their utility, with REINFORCE-based methods remaining superior due to robust pretraining.^23–25^

Inspired by Test-Time Training (TTT) in large language models, where model parameters are temporarily updated for specific tasks,^26^ we frame RL-based optimization of pretrained CLMs as a form of TTT. In LLMs, scaling inference-time sampling has shown log–linear performance improvements.^27^ Similarly, scaling TTT for CLMs—by increasing the number of RL agents or training time—offers potential to enhance chemical space exploration. Cooperative RL strategies further amplify exploration through population-based learning,^28^ though their application in molecular design remains underexplored.

In this work, we make two key contributions. First, we demonstrate that scaling TTT by increasing the number of independent RL agents follows a log–linear scaling law, significantly enhancing exploration efficiency. Conversely, extending TTT training time yields limited benefits, even with exploration bonuses or diversity filters. We also propose and evaluate cooperative RL strategies to further improve exploration efficiency. Second, we introduce MolExp, a novel benchmark to measure **Mol**ecule **Exp**loration. MolExp requires the rediscovery of structurally diverse molecules with comparable bioactivity. Unlike existing benchmarks, MolExp requires exploration of all high-reward regions of chemical space, extending the boundaries of real-world drug design.

This work provides a robust framework for enhancing chemical space exploration, addressing critical challenges in AI-driven drug discovery. By leveraging TTT and cooperative RL, we offer practical tools through MolExp and scalable strategies that can accelerate the discovery of diverse drug candidates.

Balancing the exploration-exploitation trade-off is a constant challenge in RL, including RL for drug design.^29^ Exploitation initially maximizes the reward quicker but may lead to a lower overall reward, while exploration may lead to a higher overall reward and increased diversity of solutions but takes longer. Diversity has consistently been an important topic in generative molecular design and is increasingly included in the evaluation of performance.^30,31^ Several approaches have been proposed to increase diversity, for example, using dual networks to balance exploitation-exploration during sampling,^32^ penalizing the reward of molecules similar to previously generated ones,^33^ or increasing the reward of molecules that are dissimilar to those previously generated.^34^ Recent work shows that random network distillation (RND)^35^ is particularly effective at increasing molecular diversity with low overhead due to not requiring an explicit memory of previously generated molecules.^20,36^

Previous work has applied population-based RL to de novo drug design. Hu et al.^37^ deployed up to 4 GPT agents as CLMs that use the REINVENT loss formulation with an additional penalty to discourage the *k*th agent from being similar to previous agents. This multiagent setup outperformed baseline algorithms on the GuacaMol benchmark.^13^ However, this benchmark consists of mostly simple tasks (as also stated by the authors). As demonstrated by their experiment comparing 1, 2, 4, and 8 agents all having near maximum performance and all within standard deviation. Moreover, independent agents and other cooperative strategies were not investigated. Therefore, it remains to be seen whether cooperative strategies improve performance significantly, by how much, and which cooperative strategies work best.

Generative molecular design benchmarks must reflect realistic scenarios in drug design for practical application, as gold standard evaluation via synthesis and experimental validation of molecules is too costly at scale. The GuacaMol benchmark^13^ proposed a suite of 20 tasks, the authors highlighted that ∼15/20 tasks are easily solved by the generative algorithms. The MolOpt benchmark limited these tasks to a budget of 10,000 molecules during optimization, shifting the focus to efficiency, however, this likely over-rewards exploitative algorithms as opposed to explorative ones. New criteria have been defined to additionally measure the quality of chemistry generated which has changed rank performance,^30,31^ moreover, rank performance on this MolOpt benchmark did not reflect performance on more realistic optimization objectives such as predicted binding affinity via docking.^24^ Hence, there remains significant scope for new exploration-focused objectives that better reflect realistic optimization objectives in drug design.

## Methods

### Problem Definition

The generative molecular design problem is the generation of molecules that are optimal in one or more properties defined by the drug discovery context, for example, drug-likeness and predicting binding affinity against a protein implicated in a disease. The scoring function (also referred to as an oracle) *s*(*x*) ∈ [0, 1] may combine one or more property predictors *p*_*i*_(*x*) that score a given molecule *x* returning a real value $p_{i}:X\to R$ . The final score reflects the desirability of the molecule for the given context, where the goal is to generate a molecule with the best score.

$$\begin{array}{c} \arg\;\max\;s(x)\\ x\in X \end{array}$$ 1

However, chemical space is vast, property predictors are inherently inaccurate with high uncertainty, and Pareto optimal solutions have different property profiles. Hence, multiple molecules exist across chemical space that are considered hits if the score is above a given threshold *T* (or sometimes more simply the top-*k* molecules).

$$\exists x_{i},\cdot \cdot \cdot ,x_{N}\in X,\;x_{i}\neq x_{j}\;\mathrm{such}\;\mathrm{that}\;s(x_{i})\geq T$$ 2

We propose that the problem of generative molecular design should extend to finding all possible hits across chemical space $H_{T}$ , and postulate that this better aligns with practical requirements in drug discovery.

$$H_{T}=\{x\in X|s(x)\geq T\}$$ 3

We propose that a generative model benchmark should adequately mimic this concept of identifying multiple, even structurally unrelated molecular targets in chemical space (Figure 1, right). Previously published benchmarks don’*t* test this adequately, with objectives focused on a single narrow target, or a single broad target (Figure 1, left) and performance measured only by maximum *s*(*x*) achieved. Alternative evaluations have been proposed that also measure the number of diverse hits.^30,31^ Although they are an improvement in benchmark evaluation, there are still some limitations to this approach. First, rewarding diverse hits are not suitable for many tasks converging to single solutions, hence why Renz et al. 2024 focus only on 3/23 tasks from MolOpt. Second, tasks where diverse hits are suitable, such as property prediction where multiple modes exist, are typically trivial. In some cases, maximum performance is achieved and hence no statistical differences can be observed.^37^ Lastly, when measuring the number of diverse hits it is not known how many possible diverse hits exist in solution space i.e., there is no ground truth. We propose to add a ground truth via a limited number of known modes in solution space. Moreover, ensuring the modes are distinct and structurally unrelated in a chemical sense, increases the difficulty of the benchmark and inherent exploration required, enabling the measurement of statistically significant improvements.

**Figure 1:**
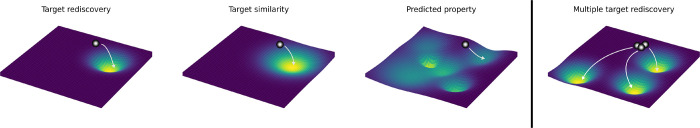
Schematic representation of a chemical space manifold according to molecular desirability, where energy wells are desirable subspaces of ‘high-reward’ areas according to a given objective. (Left) Typical objectives in the literature. (Right) Our proposal objective. The sphere represents a generative model learning to optimize desirability or maximize reward. Target rediscovery refers to a reward that reflects maximal similarity to target molecules in chemical space. Target similarity refers to a reward that reflects similarity above a certain threshold to a target molecule in chemical space, perhaps with additional multiparameter objectives. Predicted property of molecules in chemical space, where multiple regions of reward exist. Lastly, our proposal, multiple target rediscovery. involves different and distinct regions of high reward chemical space, and the goal is to rediscover all target molecules.

### Reinforcement Learning with Chemical Language Models

CLMs utilize a language representation, commonly SMILES,^9^ to leverage natural language models such as recurrent neural networks (RNNs) or GPT-style models. For example, SMILES represent the molecular structure of paracetamol with the string “CC­(=O)­Nc1ccc­(O)­cc1.” These models operate autoregressively, predicting the next token *x*_*t*_ in a sequence *X* = (*x*_1_, *x*_2_, ···, *x*_*T*_) based on the context provided by previous tokens. The process can be formalized as maximizing the conditional probability of each token given the preceding sequence *P*(*X*) = ∏ _*t* = 1_^*T*^ *P*(*x*_*t*_|*x*_<*t*_), where *x*_<*t*_ denotes the tokens up to *t* – 1. The model is trained using the cross-entropy loss function which is equivalent to the negative log-likelihood eq 4. Where *T*_*i*_ is the length of the *i*th sequence in the time domain *t*, and θ are the model parameters.

$$L=-E_{x∼D}\left[\sum_{t=1}^{T_{i}}\log(P_{\theta }(x_{t}|x_{<t}))\right]$$ 4

Reinforcement learning (RL) offers a structured approach to molecular generation by formalizing the problem as a Markov Decision Process (MDP) defined by ⟨*S*, *A*, *R*, *P*, ρ_0_⟩. Here, *S* represents the states (i.e., partial molecular structures), *A* denotes available actions (i.e., adding the next token that could describe an atom), *R* defines a scalar reward function (i.e., evaluating molecular properties), *P* is the state transition probability, and ρ_0_ is the initial state distribution. Here, the pretrained CLM acts as an already parametrized policy π_θ_ that defines an initial transition probability of selecting the next action at a given time step *P*(*s*_*t*+1_|*a*_*t*_, *s*_*t*_).

The specific RL algorithm used is a REINFORCE-based policy-gradient algorithm. REINFORCE learns a policy π_θ_ that maximizes the expected cumulative reward $J(\theta )=E_{\tau ∼\pi_{\theta }}[R(\tau )]$ , where τ represents a trajectory of states and actions. Its core objective is to adjust the policy parameters θ such that the likelihood of actions leading to higher rewards increases. This is achieved by updating θ proportionally to the gradient of the reward-weighted log-probability of action sequences, expressed as $\nabla J(\theta )=E_{\tau ∼\pi_{\theta }}\left[\sum_{t=0}^{T}\nabla_{\theta }\log\;\pi_{\theta }(a_{t}|s_{t})\cdot R(\tau )\right]$ . REINFORCE is particularly suited for problems with sparse or delayed rewards, as it treats entire trajectories as units of optimization. This works well when building molecules which only receive a reward when the building is complete.

We deviate to vanilla REINFORCE by reshaping the reward to regularize the agent policy to the trajectory probability by prior policy denoted as log π_prior_(τ) = ∑ _*t* = 1_^*T*^ log π_prior_(*x*_*t*_|*x*_<*t*_) with a coefficient σ and by adding an exponent α, as proposed by Thomas et al.^20^ shown in eq 5. Note that clipping is used to ensure the reshaped reward is always positive. These components improve the quality of molecules proposed by the agent and improve performance by steepening the gradients in the reshaped reward landscape. In addition to reshaping the reward, we implement experience replay to augment on-policy data with off-policy data at each iteration. A replay buffer is kept of high-reward molecules which is sampled with prioritization proportional to their reward. This additionally improves learning efficiency.^20,38^

$$R(\tau {)}_{\mathrm{reshaped}}=\mathrm{clip}(R(\tau )+\sigma \cdot \log\pi_{\mathrm{prior}}(\tau ){)}^{\alpha }$$ 5

### Cooperative Reinforcement Learning

Cooperative RL extends the framework of single-agent RL to systems with multiple independent or interacting agents, each learning to optimize its own policy in an environment. The problem is the same as defined before, but now with N agents.

In the independent case, each agent still aims to maximize its cumulative reward using its own $\left\langle S^{i},A^{i},R^{i},P^{i}\right\rangle$ for i∈N with an independent replay buffer for each agent. Note that independent agents will still deviate due to the stochastic nature of policy π_*i*_ sampling from the transition probabilities *P*(*s*_*t*+1_^*i*^|*a*_*t*_^*i*^, *s*_*t*_^*i*^) leading to the sampling of different actions *a*_*t*_^*i*^.

In the interaction case, we focused on methods of cooperation within a population-learning setting which typically results in more diverse solutions.^39^ In this case, using $\left\langle S,A^{i},R,P^{i}\right\rangle$ for i∈N where the states *S* and *R* may be shared across agents in a cooperative manner. We investigated the following methods of cooperation (for additional detail and equations see Section S6):

**Purge** Ensure agent replay buffers are mutually exclusive with respect to states contained within them.
**Shared** Share a single replay buffer for all agents. This should result in policy convergence and act as a type of negative control.
**Shared with bonus** Same as **Shared** but with a reward bonus for novel states not contained within the shared replay buffer.
**Noise** Apply Gaussian noise to each agent’s policy parameters at the beginning of training.
**RND** Apply RND universally to provide a reward bonus for novel states.

${\mathrm{ENT}}_{S}$ Minimize the entropy of policy uncertainty across population states to encourage specialized behavior.

${\mathrm{CE}}_{S}$ Minimize the cross-entropy loss of policy uncertainty across population states to encourage explicit specialization toward its own states.

${\mathrm{DIFF}}_{S}$ Maximize the difference between policy uncertainty in the agents own states relative to population states.

${\mathrm{DIFF}}_{N}$ Reimplementation of the cooperative strategy applied by Hu et al.^37^ which seeks to maximize the log-probability difference of on-policy states between the current agent and other agents.
**DvD** Diversity via Determinant (DvD) inspired by Parker-Holder et al.^28^ Calculate a policy behavior embedding and maximize the divergence in policy behavior as measured by the determinant of the behavioral embedding transformed by a kernel function.
**POPNORM** Normalize the agent return by subtracting the average return of the population for on-policy states, encouraging divergent behavior.

## Results

First, we describe our proposed benchmark MolExp, and show performance of various baseline RL algorithms with a comparison to an established benchmark. Then, we conducted three experiments to assess chemical exploration.

1. We investigated the effect of scaling TTT by the number of RL agents, or scaling TTT by the training time of a single RL agent by its molecule budget.
2. We proposed and tested different cooperative RL strategies to enhance exploration efficiency.
3. We translated our findings to a practically relevant task in optimizing the predicted bioactivity of generated compounds.

### Molecule Exploration Benchmark

To address our proposed problem formulation to identify *all* hits in chemical space, we designed a new molecular exploration (MolExp) benchmark. Crucially, a ground truth is required to know that *all* solutions are identified.[Fn fn1] For this reason, we use the toy-task of multiple molecule rediscovery via optimization of a similarity scoring function. To ensure relevance to real-world discovery, each set of molecules are at minimum preclinical drug candidates bioactive against the same target. Moreover, to make the objective more challenging, the set of molecules are selected to be structurally dissimilar albeit possessing similar bioactivity. This phenomena is the converse of the similarity principle.[Fn fn2] Note that activity cliffs are another example converse to the similarity principle that have been used to demonstrate more challenging scenarios for predictive models.^40,41^ To test translation of the benchmark to more practically applicable objectives, we also test chemical exploration when optimizing the predicted A2A bioactvity which we call MolExpBio.

Each run is replicated on the benchmark 5 times.

#### Oracles

The benchmark consists of four tasks. Each task is based on a set of drug candidates and their shared biological target, including antipsychotic drugs (AP), Adenosine A_2*A*_ (A2A) receptor drug candidates, beta-secretase 1 (BACE1) drug candidates, and epidermal growth factor drug candidates (EGFR). These tasks contain a set of 2, 3, 3, and 4 target molecules respectively, where the objective is to rediscover the full set of target molecules. This is similar in prospective drug discovery projects where 1 drug candidate will be progressed, but ideally with a small number of backup candidates. To mimic a chemical space where distinct regions of reward signal exist, the oracle returns the similarity to the nearest target molecule as the reward *R*(*m*) = *max*(*sim*(*m*_*i*_, *m*_*t*_1__), ···, *sim*(*m*_*i*_, *m*_*t*_*N*__)). As CLMs act in string space, we chose a string-edit similarity function based on Levenshtein distance (MolExpL). However, we additionally report the results when using fingerprint similarity (MolExp) in the Supporting Information. A more detailed description of the molecular targets chosen and the similarity function can be found in Section S2. For the MolExpBio experiment, the oracle is the predicted probability of A2A bioactivity, predicted by a Random Forest classification model trained on ECFP4 fingerprints of actives and inactives extracted from ChEMBL^42^ made available in MolScore.^43^

#### Metric

Performance is measured by calculating the product of the maximum similarity achieved to each target molecule ∏ _*t*∈*T*_ *max*(*sim*(*m*_*i*_, *m*_*t*_)). This metric focuses on the ability to generate similar molecules to all objectives. Molecular diversity is also measured by sphere exclusion diversity (SEDiv) at a sample size of 1000 ^44^ which provides a more accurate representation of chemical space coverage than internal diversity.^31,45^ Note this is equivalent to the later published #Circles^45^ metric at a Tanimoto threshold of 0.65 and normalized by the sample size.

#### Baseline Performance

We tested virtual screening, MolRL-MGPT,^37^ and multiple ACEGEN RL algorithms^20,24^ on their inherent ability for chemical exploration on the MolExpL benchmark. Note that MolRL-GPT was pretrained on the same data set for standardized comparison (see Section S1 for pretraining details). Figure 1 shows that with a budget of 10,000 the ACEGEN_*MolOpt*_ algorithm achieved the highest score, therefore, we use this RL configuration for all further experiments. However, Figure 2 illustrates that none of the tested algorithms optimize all molecular targets within the default budget (for a single replicate), and that the best scores are due to improved efficiency in maximizing similarity to a single target molecule. Therefore, there is still considerable room for improvement. These results are also observed when using fingerprint similarity oracles (see Section S4.2).

**Table 1:** Baseline Performance on the MolExpL Benchmark^a^

task	SCREEN	MolRL-MGPT	REINFORCE	REINVENT	REINVENT_*MolOpt*_	AHC	ACEGEN_*Practical*_	ACEGEN_*MolOpt*_
AP	0.53 ± 0.04	0.48 ± 0.03	0.56 ± 0.05	0.57 ± 0.04	0.63 ± 0.02	0.62 ± 0.06	**0.64** ± **0.02**	**0.64** ± **0.04**
A2A	0.38 ± 0.03	0.37 ± 0.03	0.36 ± 0.02	0.39 ± 0.08	0.38 ± 0.03	0.40 ± 0.02	0.40 ± 0.07	**0.44** ± **0.06**
BACE1	**0.29** ± **0.08**	0.20 ± 0.02	0.23 ± 0.02	0.21 ± 0.01	0.27 ± 0.03	0.23 ± 0.01	0.23 ± 0.03	0.28 ± 0.03
EGFR	0.17 ± 0.04	0.17 ± 0.01	0.15 ± 0.02	0.16 ± 0.02	0.22 ± 0.03	0.17 ± 0.03	0.17 ± 0.02	**0.2**5 ± **0.05**
Sum	1.36 ± 0.10	1.21 ± 0.05	1.30 ± 0.06	1.33 ± 0.09	1.50 ± 0.06	1.41 ± 0.07	1.44 ± 0.08	**1.62** ± **0.09**

a The ACEGEN_*MolOpt*_ RL configuration performs best in 3/4 tasks, which we carry forward for scaling experiments.

**Figure 2:**
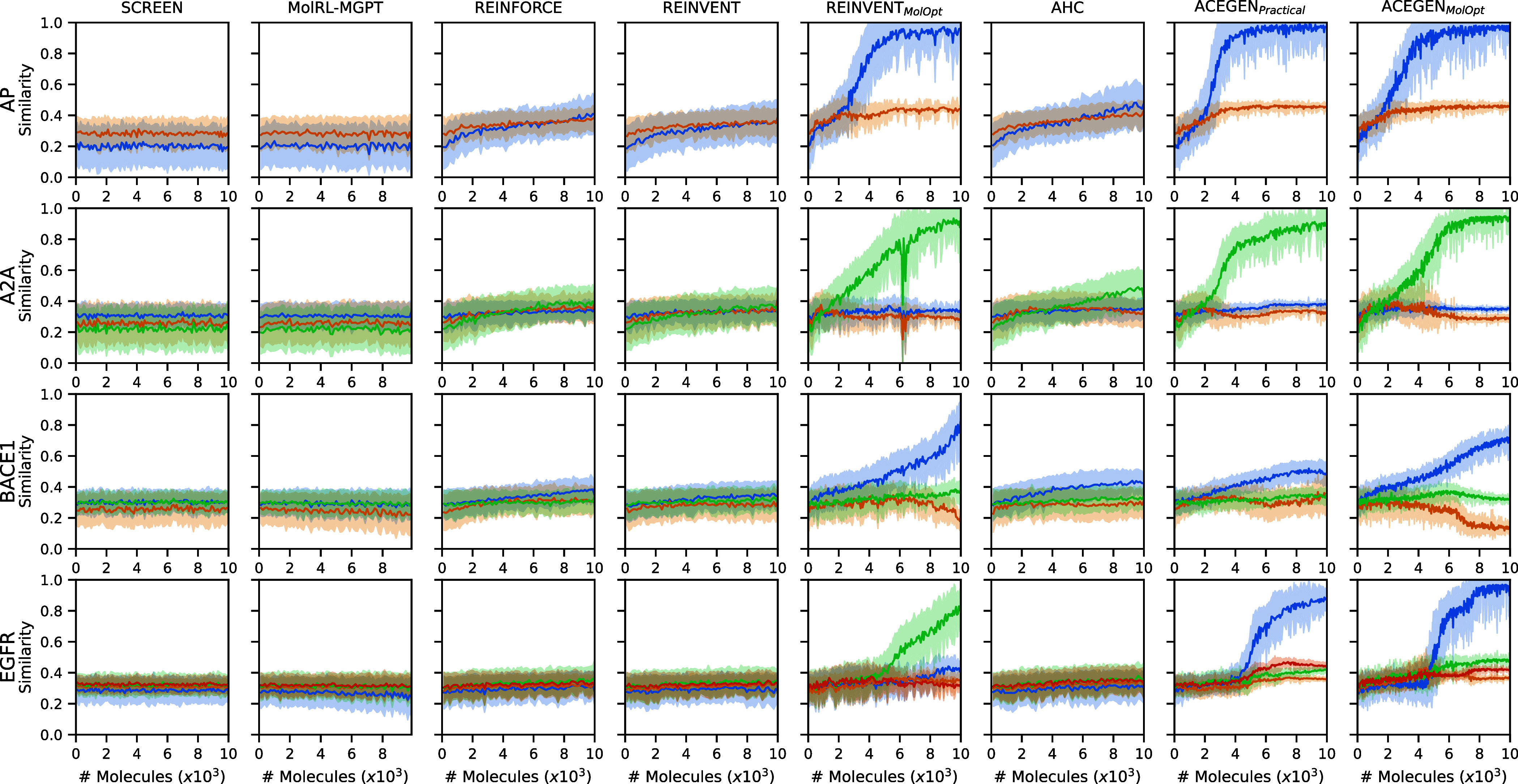
Baseline performance for each MolExpL task during RL training, single replicate. Each line color represents similarity to a target molecule. Note that ACEGEN and REINVENT_*MolOpt*_ methods outperform due to their enhanced ability to optimize similarity to at-least one target molecule. Interestingly REINVENT_*MolOpt*_ optimizes similarity to a different target molecule than ACEGEN for the EGFR task, despite identical initial policy parametrization.

#### Estimated Max

An estimated maximum performance can be calculated for ACEGEN_*MolOpt*_ RL configuration by training the agent to maximize only the similarity to each individual molecular target with the reward *R*(*m*) = *sim*(*m*_*i*_, *m*_*t*_), (see Section S4.1). This tests the agents ability to maximize similarity to each individual molecular target only, an expected prerequisite for maximizing similarity to multiple target molecules in the benchmark. Calculating the equivalent benchmark score results in an estimated maximum MolExpL score of 2.91.

#### Comparison to GuacaMol

For reference, baseline algorithms were tested on the GuacaMol benchmark also with a fixed budget of 10,000 molecules. This shows ACEGEN_*MolOpt*_ achieving the highest score (see Supporting Information). However, a key difference is that 5/6 GuacaMol similarity and rediscovery tasks are already solved by ACEGEN_*MolOpt*_ with a score ≥ 0.9, while the MolExpL benchmark results in lower scores reflecting a higher difficulty of benchmark tasks.

### Test-Time Training Scaling for Chemical Exploration

We tested if scaling TTT with larger population sizes of independent RL agents would increase chemical exploration and improve benchmark performance. In this case, we chose the best-performing RL configuration-ACEGEN_*MolOpt*_-and assigned a budget of 10,000 molecules to each agent in the population. Figure 3a shows that MolExpL performance increases log–linearly from ∼1.6 with 1 agent to ∼3.5 with 128 agents, almost reaching a maximum score of 4. Log–linear scaling was also confirmed using the vanilla REINFORCE RL configuration, albeit with lower benchmark scores. Interestingly, when the population size reached 32 the estimated maximum score for this RL configuration was exceeded, indicating that the additional variance between independent agents overcomes the additionally reward signal ambiguity introduced from multiple molecular targets. Section S5.1 shows that by increasing agents, there is more variance across the set of molecule targets. The AP task is an exception, where all 128 independent agents all maximize similarity to the same target molecule in the set. Similar results are also observed when using fingerprint similarity oracles as shown in Section S5.2. Additionally, similar scaling was achieved with up to 32 agents on the GuacaMol benchmark (Section S5.3), at which point performance on many tasks becomes saturated.

**Figure 3:**
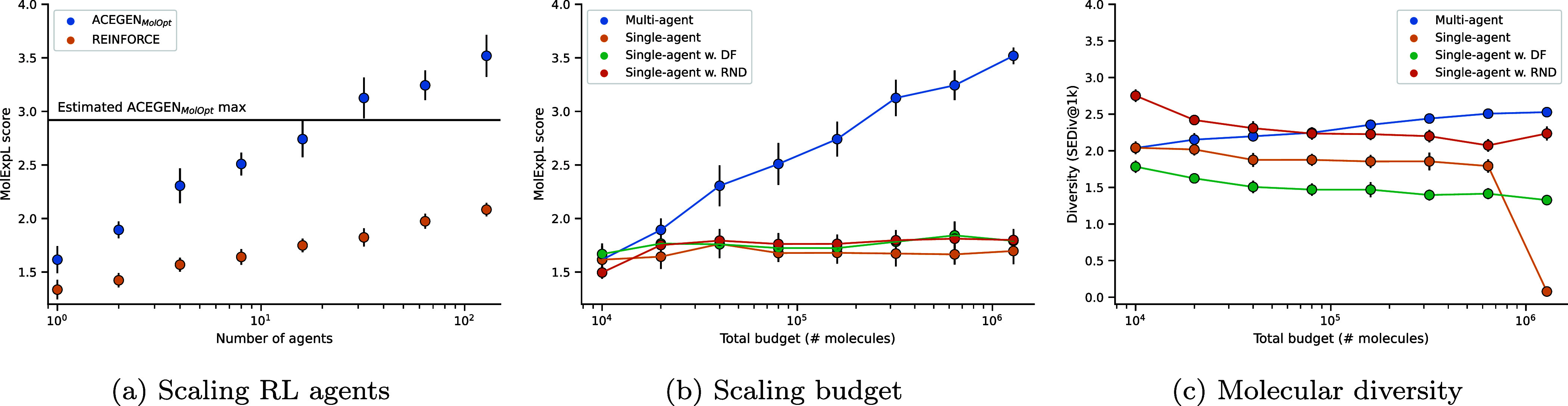
Performance on the MolExpL benchmark with scaling. (a) Scaling the number of independent ACEGEN_*MolOpt*_ and REINFORCE RL agents, each with a budget of 10,000. (b) Scaling the total budget allocated to a single agent, a single agent with an RND exploration bonus, and a single agent with a DF. (c) The diversity of sampled compounds as measured by sphere exclusion diversity.

Increasing the number of agents, each with a budget of 10,000, increases the total budget. Therefore, we compared the alternative approach of scaling TTT via increasing the budget for a single RL agent. However, this did not result in similar MolExpL performance gain as shown in Figure 3. Single RL agent performance saturates at a budget of 40,000 as the agent expectedly fails to continue exploring once one of the target molecules has been maximized (Figure S5.2). Moreover, Figure 3 shows that molecular diversity decreases while scaling budget. Employing RND as a state-of-the-art exploration strategy, the most performant exploration strategy,^20,36^ marginally improves performance and molecular diversity. Where Figure S5.3 shows that RND only results in fluctuations in similarity to one molecular target, as opposed to the desired behavior of switching from one molecular target in the set to another. We additionally tested a simple diversity filter (DF) that assigns a reward of 0 to nonunique canonical SMILES. However, the DF also failed to rescue TTT scaling in this dimension. Figure 4 exemplifies how 87 agents are required to solve the A2A task and recover all target molecules, while scaling one agent results in a focus only on the third target molecule.

**Figure 4:**
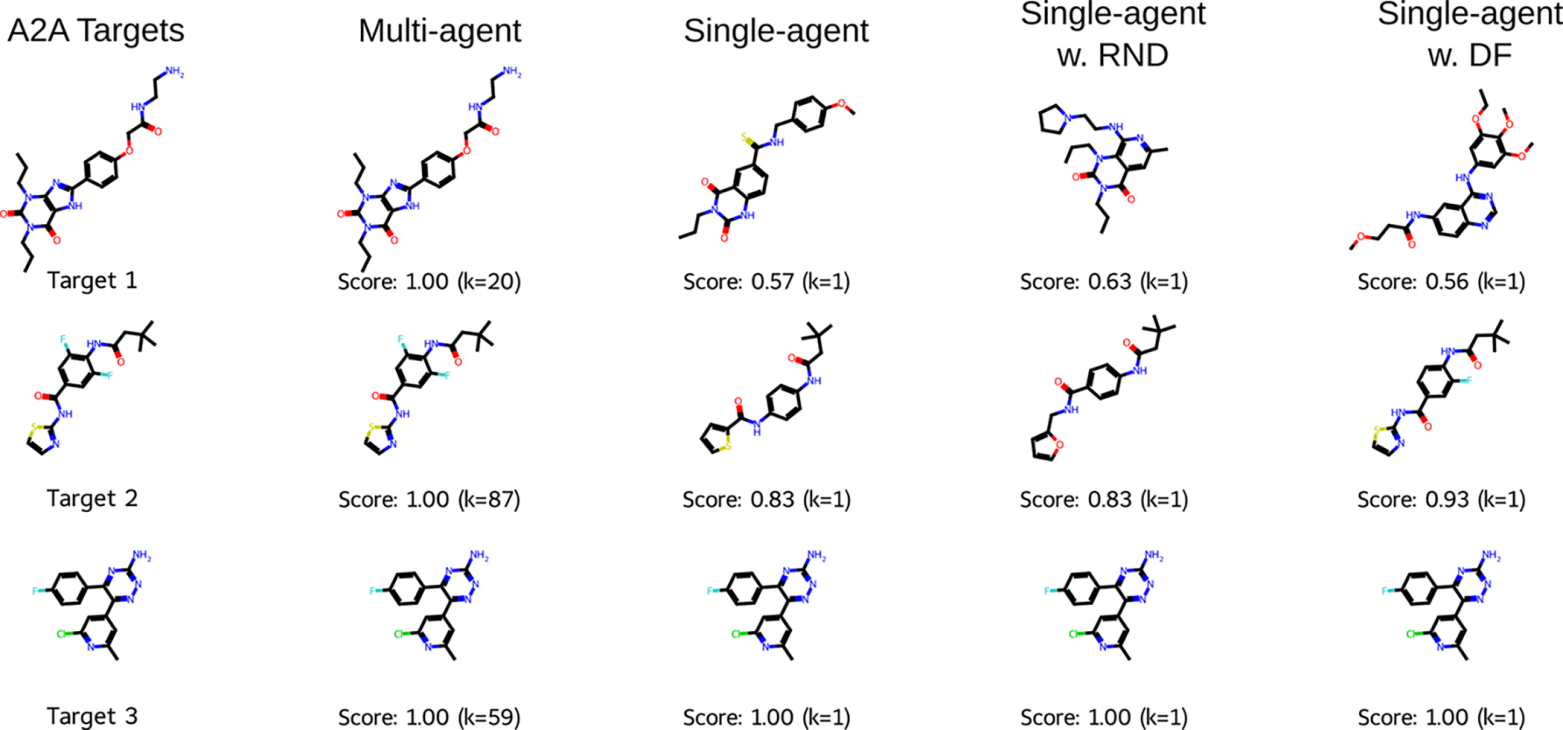
Example molecules generated compared to the set of targets in the MolExpL A2A task. The maximum similarity score and corresponding agent *k* are labeled. Note that only with 87 agents the task is solved.

### Cooperative Multiagent RL for Targeted Chemical Exploration

Cooperation within a population of RL agents can theoretically improve performance by leveraging useful information shared between agents, such as, states already visited and their corresponding reward.^39^ We explored several strategies to leverage cooperative learning to improve the efficiency of chemical exploration. Considering the maximum number of molecular targets in a task set was 4, we investigated strategies utilizing 4 RL agents. Where, the best-case scenario is each agent maximizing similarity to a different molecular target in the set.

We investigated an extensive range of the different cooperative strategies on the MolExpL benchmark, including the MolRL-MGPT baseline with the same budget and number of agents, shown in Figure 5a. All of the strategies tested were similar or worse in performance, with the best being POPNORM, ${\mathrm{ENT}}_{S}$ , and ${\mathrm{DIFF}}_{N}$ . However, no strategy significantly outperformed independent agents as measured by a Bonferroni-corrected, one-tailed *t* test. On the other hand, Figure 5b shows that many cooperative strategies successfully resulted in corresponding increases in molecular diversity, namely RND, ${\mathrm{ENT}}_{S}$ , ${\mathrm{CE}}_{S}$ , and ${\mathrm{DIFF}}_{S}$ . This is usually anticorrelated with MolExpL score. This highlights the difficulty and complexity in achieving targeted exploration, where additional diversity appears to hinder learning, or is naive rather than targeted on favorable regions of chemical space. These trends were even more prominent when using fingerprint similarity oracles (see Section S7.2).

**Figure 5:**
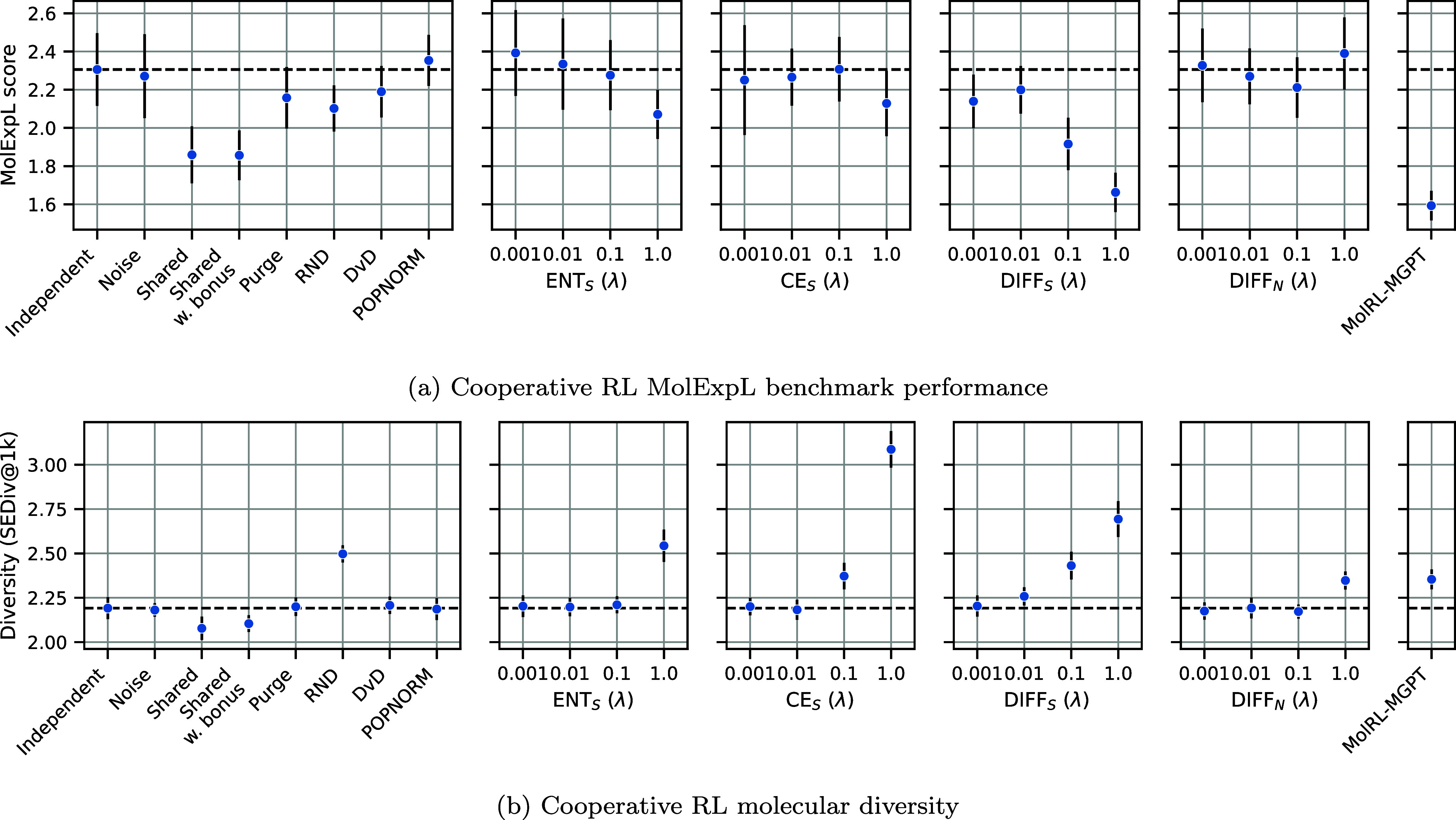
Performance comparison of (a) MolExpL Score and (b) molecular diversity of different 4-agent cooperative strategies on the MolExpL benchmark, each with a budget of 10,000. The dashed line represents the average of 4 independent agents as the baseline.

Assessing the example behavior of different agents shows that some cooperative strategies do result in divergent behavior, but to the detriment of learning efficiency by slowing or inhibiting the learning of subsequent agents in a population (see Section S7). It is hypothesized that this is due to the intra-agent “repellent” nature of many of the cooperative strategies tested–especially during the early stages of optimization when no high reward region has been found yet.

### Maximizing Predicted A2A Bioactivity

To represent a practically applicable objective function, we additionally tested TTT scaling behavior when maximizing the predicted probability of A2A bioactivity as measured by the QSAR classification model from MolScore.^43^ Increasing chemical exploration during optimization should increase the probability of recovering the set of known A2A drug candidates used in the MolExp A2A task, two of which are present in the training data set positive class. Therefore, we use the same metric to measure the maximum similarity achieved to the A2A target molecule set, we refer to this additional experiment as MolExpBio. However, this now depends on the properties of the QSAR model and therefore, is not theoretically guaranteed.

Figure 6a shows that scaling the number of independent RL agents increases the benchmark score, again more than scaling the budget of a single RL agent (Figure 6b). In other words, scaling RL agents increases the maximum similarity to the set of target molecules, and hence the chance of rediscovering the drug candidates. This represents increased chemical exploration of the reward landscape. This task highlights the translational benefit of TTT scaling with RL agents to real-world drug design objectives.

**Figure 6:**
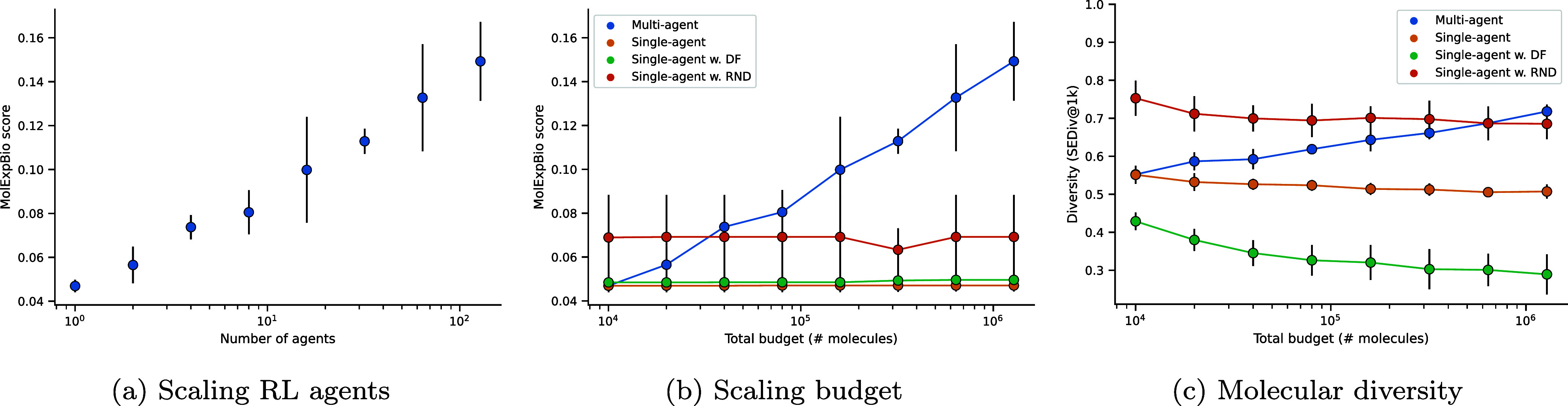
Performance on the MolExpBio task with scaling. (a) Scaling the number of independent ACEGEN_*MolOpt*_ and REINFORCE RL agents, each with a budget of 10,000. (b) Scaling the total budget allocated to a single agent, a single agent with an RND exploration bonus, and a single agent with a DF. (c) The diversity of sampled compounds as measured by sphere exclusion diversity.

## Conclusions

In this paper, we probe the exploration of chemical space with a CLM to identify multiple candidate molecules in the context of drug discovery. In this effort, we introduce MolExp as a new benchmark, showing poor intrinsic exploration of current baseline algorithms. We showed that scaling a population of independent RL agents was the best approach to increasing benchmark performance, practically solving it with 128 agents. This included comparisons to scaling budgets for single RL agents and an extensive range of cooperative strategies.

Moreover, we found that performance improved log–linearly, similarly observed with test-time inference scaling of LLMs. In this regard, training the pretrained CLM agent with RL on a specific test-time task (each of the four benchmark tasks) can be viewed as TTT. We than scale the whole TTT process by increasing the number of RL agents, resulting in similar scaling laws observed with test-time inference scaling in LLMs.

Overall, this work takes an important step toward identifying *all* possible molecules of interest in drug-like chemical space. Our MolExp benchmark is the first to explicitly ask this question, which we hope will guide algorithmic improvement until chemical space can be efficiently and thoroughly explored for a given context. TTT scaling through populations of RL agents are a promising solution toward this future.

### Limitations

The similarity functions used for the tasks are unlikely to be used in real-world drug design. In practice, predictive models of bioactivity, binding affinity, and ADMET properties are more common. However, the set of *all* the best possible solutions for such scoring functions (e.g., molecular docking) are currently unknowable. For this reason, MolExp­(L) implements similarity functions as perfect scoring functions and tasks with known ground truths. We acknowledge this difference and strengthen the translational assumption with similar empirical results on other established benchmarks and a real-world objective in A2A bioactivity prediction.

We did not investigate the effect of model architecture and training data set in this work, which has been thoroughly investigated elsewhere.^12,46^ Instead we focus on maximizing chemical exploration efficiency given the same or similar starting policy. These results are expected to translate to any starting policy.

The caveat of scaling TTT with more RL agents or increased budget is increased walltime requirements, as shown in Section S9. This reiterates the importance of trying to use cooperative methods to improve exploration efficiency, as well as, the use of this benchmark and results as a tool for further research into targeted and efficient chemical space exploration.

## Supplementary Material



## Data Availability

All code is openly available under an MIT license. The CLM-based RL agents were implemented in ACEGEN, available on GitHub athttps://github.com/Acellera/acegen-open, while the benchmark was implemented in MolScore available on GitHub at https://github.com/MorganCThomas/MolScore or in the Python Package Index https://pypi.org/project/MolScore/. Note the MolExp pretraining data set is also available in the MolScore repository.

## Footnotes

1 Oracles that are more practically relevant, such as bioactivity prediction or docking, do not contain a ground truth set of all possible high scoring solutions.

2 In drug discovery, the similarity principle states that similar molecules *Sim*(*m*_1_, *m*_2_) ≥ ϵ possess similar bioactivity |*f*(*m*_1_) – *f*(*m*_2_)| ≤ δ. However, this does not prove the converse and many exceptions exist that are more difficult to model.

## Notes

The Appendices referred to throughout the text (PDF)
