# Supplementary material for: Test-Time Training Scaling Laws for Chemical Exploration in Drug Design
Source: J Chem Inf Model. 2025 Dec 9;65(24):13178–86. doi: 10.1021/acs.jcim.5c02316 (PMC12728925; doi:10.1021/acs.jcim.5c02316)
Supplement: Supplementary file 1 [file ci5c02316_si_001.pdf]

Supporting Information: Test-Time Training Scaling Laws for Chemical Exploration in Drug Design

Morgan Thomas,[*,†,‡] Albert Bou,[†] and Gianni De Fabritiis[*,¶,†,§]

†*Computational Science Laboratory, Universitat Pompeu Fabra, Barcelona Biomedical Research Park (PRBB), C Dr. Aiguader 88, 08003 Barcelona, Spain*

‡*Department of Medical Sciences, Khalifa University of Science and Technology, 127788 Abu Dhabi, UAE*

¶*Institució Catalana de Recerca i Estudis Avançats (ICREA), Passeig Lluis Companys 23, 08010 Barcelona, Spain*

§*Acellera Labs, C Dr Trueta 183, 08005, Barcelona, Spain*

E-mail: morganthomas263@gmail.com; g.defabritiis@gmail.com

Contents

1 Model pre-training **S-3**

1.1 Datasets S-3

1.2 ACEGEN S-3

1.3 MolRL-MGPT S-4

2 Molecular exploration benchmark **S-5**

3 Baseline algorithms **S-8**

4 Baseline performance S-14
4.1 MolExpL . . . S-14
4.2 MolExp . . . S-15
4.3 GuacaMol . . . S-17
5 TTT scaling S-19
5.1 MolExpL . . . S-19
5.2 MolExp . . . S-23
5.3 GuacaMol . . . S-25
6 Cooperative strategies S-27
7 Cooperative RL S-32
7.1 MolExpL . . . S-32
7.2 MolExp . . . S-36
8 A2A bioactivity maximization S-41
9 Computational resource S-44
References S-45

1 Model pre-training

1.1 Datasets

MolExp benchmark The pre-training dataset for the MolExp(L) benchmark was curated from the ChEMBL34 database.[S1] First, the database was filtered to ensure no molecules existed with more 10 rotatable bonds, a logP above 5.5, a molecular weight outside of 150-650 Da, or contained atoms not in a comment set $a \in C, N, S, O, F, Cl, Br, H$. All molecules were neutralized if possible, converted to non-isomeric SMILES (i.e., containing no stereoinformation) and de-duplicated. This resulted in a dataset of 1,711,022 unique molecules. Finally, the target molecules of the MolExp benchmark section 2 were purposefully confirmed to be present within the training dataset, to ensure that objectives were theoretically achievable.

GuacaMol benchmark The pre-training dataset for the GuacaMol[S2] benchmark was taken directly from the original publication without any further processing. This dataset was downloaded from `https://figshare.com/projects/GuacaMol/56639`.

1.2 ACEGEN

MolExp benchmark The CLM constituting the prior policy is a gated recurrent unit (GRU) network with an embedding of 256 and 3 layers of GRU cells with hidden dimension 512. This was trained with restricted SMILES randomization[S3] for 10 epochs with a batch size of 128. This was implemented such that a random SMILES string augmentation was applied each time a dataset molecule was sampled during training. An ADAM optimizer was employed with a learning rate of 0.001 and step scheduler that dropped the learning rate every 500 steps. The learning rate, training loss, and SMILES validity during training is shown in Figure 1.1.

GuacaMol benchmark The CLM constituting the prior policy is an long short-term memory (LSTM) network with the same hyperparameters as the model used in GuacaMol benchmark. This includes an embedding of 1024 and 3 layers of LSTM cells with hidden

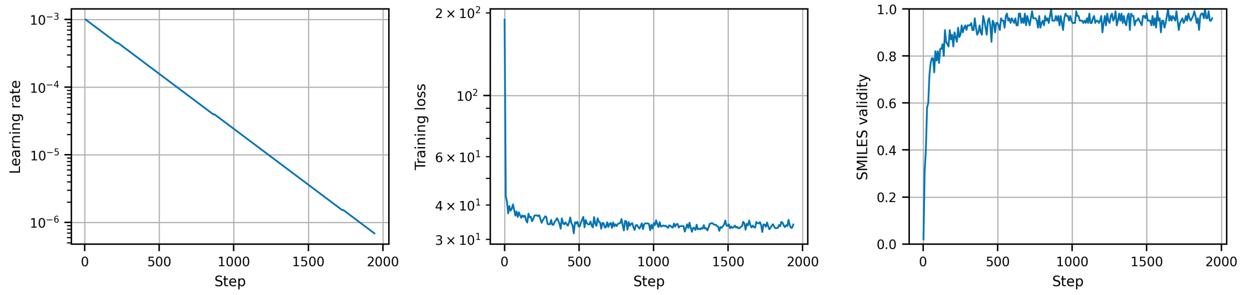

Figure 1.1: Pre-training of the GRU CLM on the MolExp benchmark pre-training dataset curated from ChEMBL34.

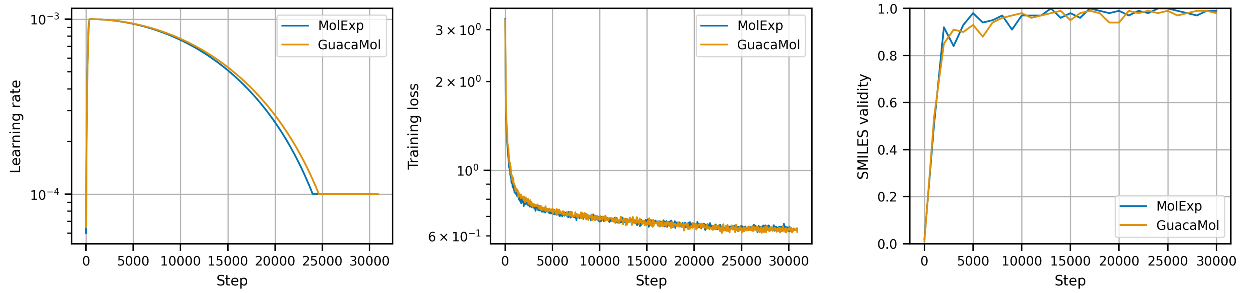

Figure 1.2: Pre-training of the MolRL-MGPT CLM on the MolExp benchmark pre-training dataset and GuacaMol benchmark pre-training dataset.

dimension 1024, as well as a dropout rate of 0.2. The pre-trained weights were loaded from the GuacaMol benchmark resulting in the exact same prior and initial policy.

1.3 MolRL-MGPT

The MolRL-MGPT model was re-implemented following the code provided from the original publication.[S4] This CLM constituting the prior policy is a GPT-style Transformer model with 8 layers each with 8 attention heads. For a standardized comparison, the model was pre-trained on the benchmark training datasets respectively. For hardware reasons the batch size was lowered from 2048 to 512, with all other hyperparameters kept equal including training for 10 epochs. The learning rate, training loss, and SMILES validity during training is shown in Figure 1.2.

2 Molecular exploration benchmark

We selected four tasks, each with 2-4 objectives as molecular targets to be rediscovered based on real-world drug candidates ranging from pre-clinical (PC), clinical phases 1-3 (PH I-III) and approved (PH IV), shown in Figure 2.1 to Figure 2.4. For each task, the goal is to maximize the reward $R(m)$ for a given molecule m, where the reward is similarity to the closest molecular target m_t:

$$R(m) = max(sim(m_i, m_{t_1}), ..., sim(m_i, m_{t_N})) \tag{2.1}$$

In this work, the similarity function sim is implemented as the Levenshtein similarity between the canonical SMILES strings (MolExpL). This was chosen because the CLM generates and acts in a SMILES environment. The Levenshtein similarity function was created by normalizing the Levenshtein distance by the length of the reference string, clipped to ensure the output value was bound between [0, 1]:

$$sim_{\mathbb{L}}(m, m_t) = clip(\frac{1 - dis_{\mathbb{L}}(m, m_t)}{|m_t|}) \tag{2.2}$$

Note that the SMILES strings of both m m_t are canonicalized and both used non-isomeric SMILES. Moreover,we made an alternative similarity function available using the Tanimoto similarity of ECFP fingerprints for application with non-language models (MolExp), although the Levenshtein similarity will still work provided a SMILES representation is given.

Performance was evaluated based on the ability to generate molecules close to all molecular targets by taking the product:

$$\prod_{t \in T} \frac{\sum_{i \in N} sim(m_i, m_t)}{N} \tag{2.3}$$

This formulation tests the generative models ability to maximize the reward and remain intrinsically curious and continue exploring - even when the expected reward may already

be high.

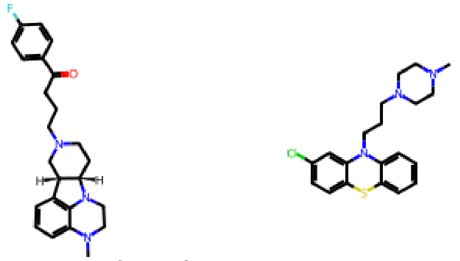

LUMATEPERONE (PH IV) PROCHLORPERAZINE (PH IV)

Figure 2.1: Antipsychotic (AP) drugs as molecular targets. Both are clinically approved antipsychotics, however, Lumateperone was approved in 1956, while Prochlorperazine was the most recently approved in 2019.

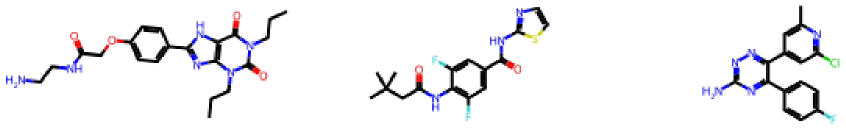

XAC (PC) LUAA47070 (PC) IMARADENANT (PH II)

Figure 2.2: Adenosine A_{2A} (A2A) receptor drug candidates as molecular targets. All the molecular candidates are A_{2A} receptor antagonists that bind to the same orthosteric site but with alternative binding modes. XAC binds from N253 up towards the extracellular side with a polar tail that extends into solvent, LUAA47070 binds via a water mediated interaction with N253 (note we use active metabolite, desphospho-structure), while Imaradenant binds from N253 down towards the intrahelical bundle occupying lipophilic hotspots.

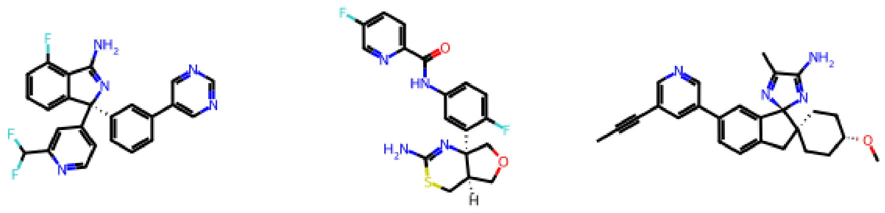

AZD-3839 (PH I) LY-2886721 (PH I) LANABECESTAT (PH III)

Figure 2.3: Beta-secretase I (BACE1) receptor drug candidates as molecular targets. All are high-affinity inhibitors of BACE1 receptor with a common functional amidine/hiourea group to bind to the dual Asparctic acids in the pocket but are otherwise topologically different.

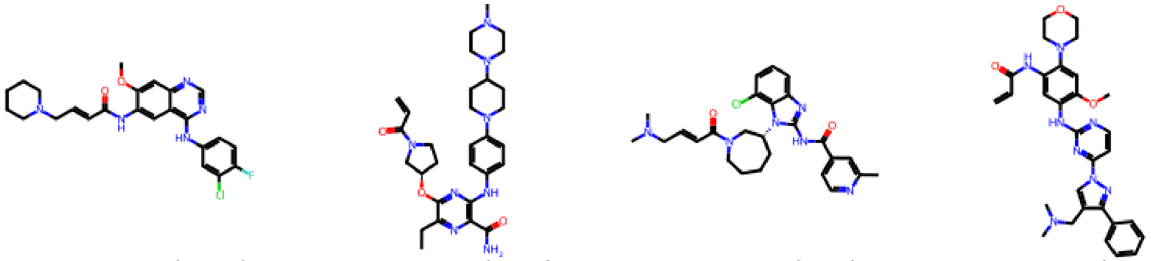

DACOMITINIB (PH IV) NAQUOTINIB (PH III) NAZARTINIB (PH II) LAZERTINIB (PH III)

Figure 2.4: Epidermal growth factor (EGFR) drug candidates as molecular targets. All molecules are high-affinity covalent inhibitors of EGFR, sharing the reactive acrylamide functional group to bind to cysteine. Otherwise they are the four most topologically diverse molecules out of the 13 sub-100nM activity, at least phase I EGFR inhibitors found in ChEMBL34.

3 Baseline algorithms

A description of baseline algorithms and in particular their update step are detailed below, followed by the hyperparameters for each algorithm, followed by a pseudo-algorithm describing the general RL rollout and update procedure for these single-agent baseline algorithms 1. Note this algorithm does not apply to MolRL-MGPT which is a multi-agent baseline.

SCREEN: This virtual screening baseline iteratively samples without replacement from the CLM training dataset section 1 curated from ChEMBL34. Note that all the target molecules from MolExp benchmark are included in the dataset, therefore, it is possible to achieve a perfect score if they are sampled. However, this is unlikely given the budget of 10,000 molecules out of the possible 1.7M.

REINFORCE: This baseline was implemented as the vanilla reinforce algorithm where the objective is to maximize the likelihood of sequences weighted by the cumulative reward $R(\tau)$. In this implementation we additionally applied experience replay to augment on-policy data with previously high rewarded molecules. All configuration parameters are shown in Table 3.1.

$$\nabla J(\theta) = \mathbb{E}_{\tau \sim \pi_\theta} \left[\sum_{t=0}^{T} \nabla_\theta \log \pi_\theta(a_t|s_t) \cdot R(\tau) \right] \tag{3.1}$$

REINVENT: This baseline was implemented following the REINVENT formulation[S5] whereby a fixed version of the prior policy π_{prior} (i.e., the pre-trained CLM) is used to regularize the updates of the agent policy π_{agent} being trained. This is a form of reward shaping as shown in following Equation 3.2. This also introduces a hyperparameter σ that determines the balance between $\pi_{prior} - \pi_{agent}$ difference or the reward $R(\tau)$. The hyperparameters used corresponding to the default frequently used in the literature are shown in Table 3.2.

$$R(\tau)_{reshaped} = \frac{(\pi_{prior} - \pi_{agent} + \sigma \cdot R(\tau))^2}{\pi_{agent}} \tag{3.2}$$

REINVENT$_{MolOpt}$: This baseline is a hyperparameter optimized configuration of REIN-

VENT for performance on the MolOpt benchmark. Notably this increases σ to focus more on molecule reward higher than regularization to the prior and increases the amount of experience replay used. All configuration parameter are shown in Table 3.3.

AHC: Augmented Hill-Climb[S6] is a variation of REINVENT that conducts on-policy hill-climbing, i.e., on-policy molecules are ranked by reward $R(\tau)$ and only the top-k molecules are retained. This shift focuses learning on high-reward molecules which improves efficiency, and helps to avoid too much regularization in low-reward scenarios. All configuration parameters are shown in Table 3.4.

MolRL-MGPT: MolRL-MGPT is a cooperative multi-agent RL baseline with 4 GPT-style CLM agents. This follows the REINVENT formulation with the addition of a second loss term for the k-th agent encouraging reward scaled deviation of the current agent to previous ones. This baseline was implemented using the code provided alongside the publication.[S4] The default hyperparameters provided with the code were used, as there were insufficient details for full reproducibility of paper results.

$$\mathcal{L}^{k}_{DIFF_{\mathcal{N}}} = -\sigma_2 \cdot \sum_{i=1}^{k-1} R(\tau) \cdot |\log \pi_k(\tau) - \log \pi_i(\tau)| \tag{3.3}$$

Table 3.1: Hyperparameters for REINFORCE.

Hyperparameter	Value
num_envs	128
total_smiles	10,000
model	GRU (embedding of size 256 + 3 layer GRU of size 512 + MLP)
lr	0.0001
experience_replay	True
replay_buffer_size	100
replay_batch_size	10

Table 3.2: Hyperparameters for REINVENT.

Hyperparameter	Value
num_envs	128
total_smiles	10,000
model	GRU (embedding of size 256 + 3 layer GRU of size 512 + MLP)
lr	0.0001
experience_replay	True
replay_buffer_size	100
replay_batch_size	10
sigma	120

Table 3.3: Hyperparameters for REINVENT$_{MolOpt}$.

Hyperparameter	Value
num_envs	64
total_smiles	10,000
model	GRU (embedding of size 256 + 3 layer GRU of size 512 + MLP)
lr	0.0005
experience_replay	True
replay_buffer_size	100
replay_batch_size	24
sigma	500

Table 3.4: Hyperparameters for AHC.

Hyperparameter	Value
num_envs	128
total_smiles	10,000
model	GRU (embedding of size 256 + 3 layer GRU of size 512 + MLP)
lr	0.0001
experience_replay	True
replay_buffer_size	100
replay_batch_size	10
sigma	60
topk	0.5

Table 3.5: Hyperparameters for $\text{ACEGEN}_{Practical}$.

Hyperparameter	Value
num_envs	128
total_smiles	10,000
model	GRU (embedding of size 256 + 3 layer GRU of size 512 + MLP)
lr	0.0001
experience_replay	True
replay_batch_size	10
replay_buffer_size	100
replay_sampler	uniform
sigma	0.005
topk	0.5
alpha	5
baseline	mab

Table 3.6: Hyperparameters for ACEGEN_{MolOpt}.

Hyperparameter	Value
num_envs	32
total_smiles	10,000
model	GRU (embedding of size 256 + 3 layer GRU of size 512 + MLP)
lr	0.0001
experience_replay	True
replay_batch_size	50
replay_buffer_size	100
replay_sampler	prioritized
sigma	0.001
topk	0.5
alpha	3
baseline	False

Table 3.7: Hyperparameters for MolRL-MGPT.

Hyperparameter	Value
num_envs	128
total_smiles	10,000
model	GPT (embedding of size 256 + 8 layer with 8 attention heads + MLP)
lr	0.0001
experience_replay	True
replay_batch_size	5
replay_buffer_size	25
replay_sampler	uniform
$sigma_1$	100
$sigma_2$	0.5

Algorithm 1: Single-Agent RL

Input: Stochastic policy π_θ, horizon T, replay buffer $\mathcal{B}$, batch size B, (optional) sigma value σ

Initialize pretrained policy π_θ

Initialize replay buffer $\mathcal{B} \leftarrow \varnothing$

Set counter $N \leftarrow 0$ `// total molecules sampled so far`

(Optional) Initialize frozen prior policy $\pi_{\text{prior}} \leftarrow \pi_\theta$

while $N < M$ **do**

  `// (1) Rollout: generate on-policy batch`

  for $b = 1$ **to** B **do**

    $s_0 \leftarrow$ `BOS`

    for $t = 0$ **to** $T - 1$ **do**

      Sample action $a_t \sim \pi_\theta(\cdot \mid s_t)$

      Update state $s_{t+1} \leftarrow \text{Append}(s_t, a_t)$

      if $a_t =$ *`EOS`* **then**

        break

      end

    end

    Store trajectory $\tau^{(b)} = (s_0, a_0, \ldots, s_T, a_T)$

  end

  Denote $\tau_{\text{on}}^B = \{\tau^{(b)}\}_{b=1}^B$

  Update counter $N \leftarrow N + |\tau_{\text{on}}^B|$

  `// (2) Augment with replay buffer samples`

  Sample $\tau_{\text{off}} \sim \mathcal{B}$

  Form combined batch $\tau^B \leftarrow \tau_{\text{on}}^B \cup \tau_{\text{off}}$

  `// (3) Compute rewards via oracle`

  $R(\tau^B) \leftarrow f(\tau^B)$ `// May include DF penalty or RND bonus`

  `// (4) Policy update using chosen method` Ψ

  $\pi_\theta \leftarrow \text{Update}_\Psi(\pi_\theta, \tau^B, R(\tau^B), [\pi_{\text{prior}}], [\sigma])$

  `// (5) Update replay buffer with top-performing trajectories`

  $\mathcal{B} \leftarrow \mathcal{B} \cup \text{Top-}k(\tau^B, R(\tau^B))$

end

4 Baseline performance

4.1 MolExpL

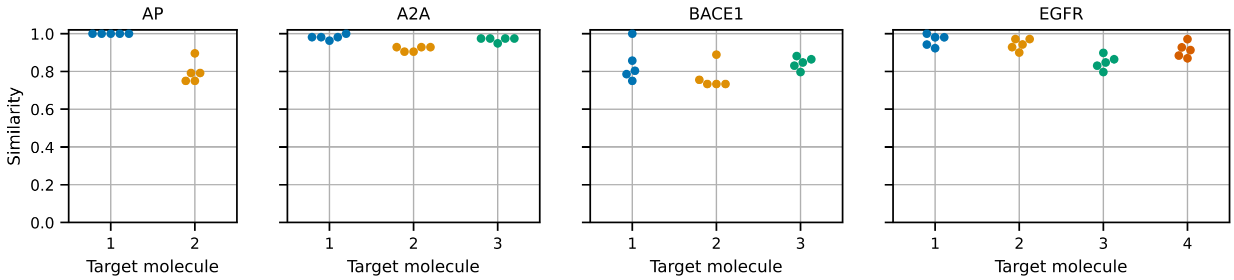

Figure 4.1: Estimated maximum performance achieved by ACEGEN$_{MolOpt}$. ACEGEN$_{MolOpt}$ was trained to maximize the similarity to each individual molecular target separately for a budget of 10,000 molecules. Based on this, the estimated maximum performance on the benchmark is 2.92.

4.2 MolExp

Table 4.1: Baseline performance on the MolExp benchmark.

	SCREEN	MolRL-MGPT	REINFORCE	REINVENT	REINVENT$_{MolOpt}$	AHC	ACEGEN$_{Practical}$	ACEGEN$_{MolOpt}$
AP	0.32 ± 0.10	0.24 ± 0.05	0.37 ± 0.02	0.25 ± 0.04	0.45 ± 0.03	**0.47 ± 0.04**	0.40 ± 0.02	0.42 ± 0.04
A2A	0.08 ± 0.02	0.08 ± 0.01	0.09 ± 0.01	0.06 ± 0.01	0.12 ± 0.02	0.11 ± 0.03	**0.13 ± 0.03**	**0.13 ± 0.05**
BACE1	**0.16 ± 0.07**	0.07 ± 0.01	0.05 ± 0.01	0.05 ± 0.01	0.06 ± 0.01	0.06 ± 0.01	0.06 ± 0.01	0.06 ± 0.01
EGFR	0.04 ± 0.01	0.03 ± 0.01	0.04 ± 0.01	0.03 ± 0.00	0.05 ± 0.02	0.04 ± 0.01	0.05 ± 0.02	**0.06 ± 0.01**
Sum	0.60 ± 0.12	0.42 ± 0.05	0.55 ± 0.02	0.39 ± 0.04	0.68 ± 0.05	0.68 ± 0.05	0.64 ± 0.04	**1.07 ± 0.08**

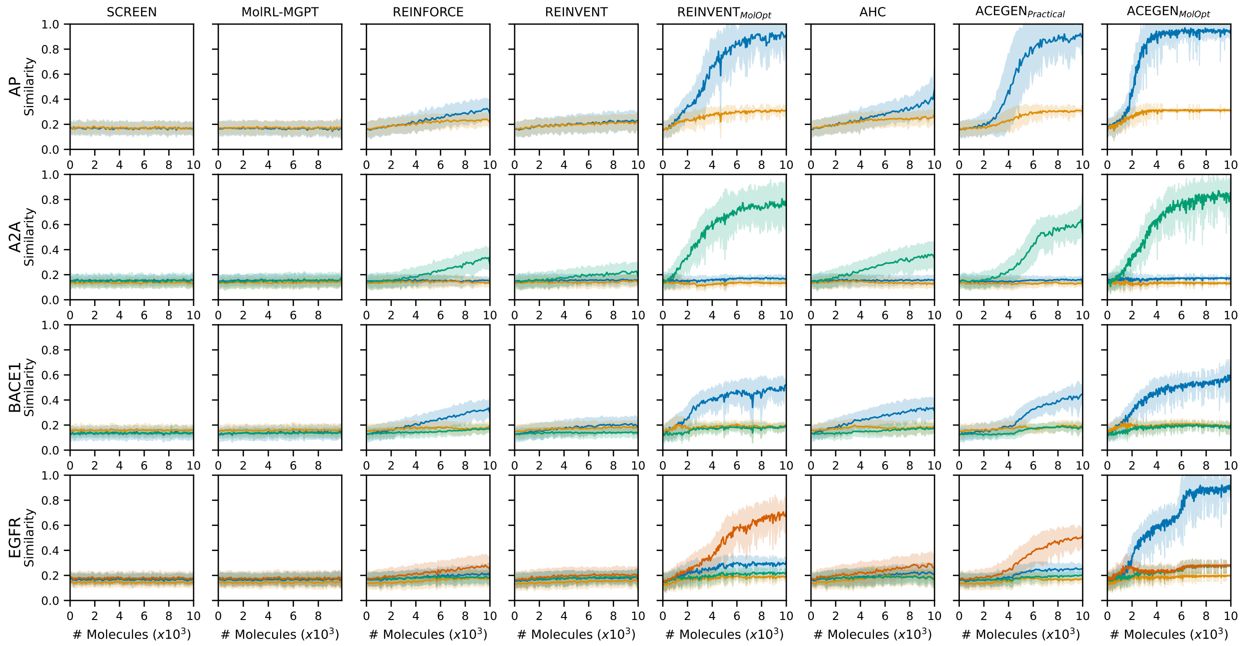

Figure 4.2: Baseline performance for each MolExp task during RL training, single replicate. Each line color represents similarity to a target molecule. Note that ACEGEN and REINVENT$_{MolOpt}$ methods outperform due to their enhanced ability to optimize similarity to at-least one target molecule.

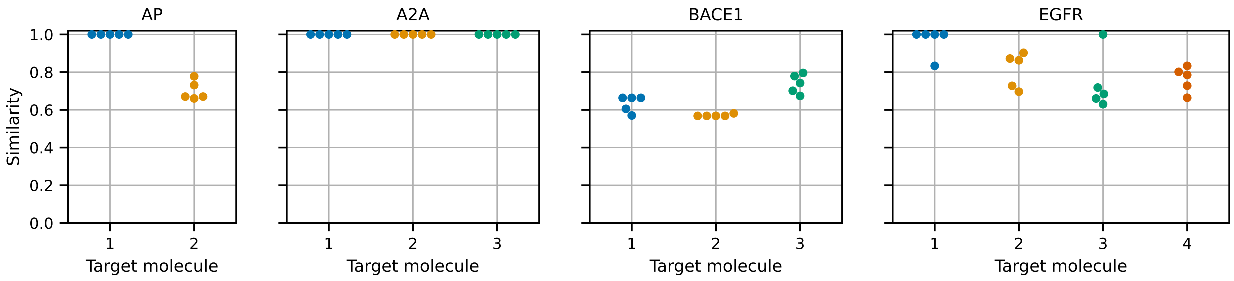

Figure 4.3: Estimated maximum performance achieved by ACEGEN$_{MolOpt}$. ACEGEN$_{MolOpt}$ was trained to maximize the similarity to each individual molecular target separately for a budget of 10,000 molecules. Based on this, the estimated maximum performance on the benchmark is 2.41.

4.3 GuacaMol

Baseline algorithms were run on the GuacaMol benchmark also with 5 replicates, and for a standardized comparison, a budget constraint of 10,000 molecules. Previously published benchmark values have no restriction on budget, therefore, varying budgets have been used per algorithm. For this reason, values will differ from those previously published.

Table 4.2: Baseline performance on the GuacaMol benchmark, part I.

Task	SMILES LSTM	SMILES GA	Graph GA	Graph MCTS	Frag GT	SCREEN
Albuterol similarity	0.92 ± 0.02	0.45 ± 0.05	0.91 ± 0.05	0.59 ± 0.03	0.99 ± 0.00	0.53 ± 0.01
Amlodipine MPO	0.65 ± 0.02	0.52 ± 0.05	0.65 ± 0.04	0.44 ± 0.01	0.69 ± 0.04	0.56 ± 0.03
Aripiprazole similarity	0.81 ± 0.03	0.37 ± 0.04	0.83 ± 0.10	0.25 ± 0.01	0.87 ± 0.06	0.50 ± 0.00
C11H24	0.59 ± 0.05	0.01 ± 0.01	0.63 ± 0.16	0.06 ± 0.01	0.81 ± 0.02	0.05 ± 0.01
C9H10N2O2PF2Cl	0.60 ± 0.01	0.14 ± 0.06	0.67 ± 0.10	0.56 ± 0.02	0.77 ± 0.03	0.39 ± 0.01
Celecoxib rediscovery	**1.00 ± 0.00**	0.30 ± 0.01	0.70 ± 0.12	0.29 ± 0.03	0.70 ± 0.07	0.44 ± 0.03
Deco hop	0.66 ± 0.01	0.59 ± 0.01	0.65 ± 0.02	0.55 ± 0.00	**0.73 ± 0.01**	0.62 ± 0.01
Fexofenadine MPO	0.76 ± 0.01	0.67 ± 0.02	0.79 ± 0.01	0.58 ± 0.00	0.83 ± 0.02	0.72 ± 0.02
Median molecules 1	0.34 ± 0.00	0.14 ± 0.02	0.28 ± 0.01	0.18 ± 0.01	0.28 ± 0.02	0.20 ± 0.01
Median molecules 2	0.30 ± 0.01	0.18 ± 0.01	0.28 ± 0.02	0.13 ± 0.00	0.33 ± 0.06	0.23 ± 0.01
Mestranol similarity	0.68 ± 0.05	0.32 ± 0.04	0.64 ± 0.11	0.27 ± 0.01	0.57 ± 0.02	0.48 ± 0.03
Osimertinib MPO	0.83 ± 0.01	0.77 ± 0.02	0.83 ± 0.01	0.71 ± 0.01	**0.87 ± 0.01**	0.79 ± 0.00
Perindopril MPO	0.51 ± 0.01	0.41 ± 0.03	0.53 ± 0.03	0.28 ± 0.01	0.58 ± 0.02	0.47 ± 0.02
Ranolazine MPO	0.77 ± 0.01	0.55 ± 0.02	0.72 ± 0.02	0.24 ± 0.04	**0.91 ± 0.01**	0.71 ± 0.01
Scaffold hop	0.58 ± 0.01	0.46 ± 0.01	0.58 ± 0.02	0.42 ± 0.00	0.77 ± 0.12	0.51 ± 0.02
Sitagliptin MPO	0.34 ± 0.03	0.22 ± 0.03	**0.49 ± 0.08**	0.21 ± 0.02	0.45 ± 0.03	0.30 ± 0.02
Thiothixene rediscovery	0.64 ± 0.04	0.30 ± 0.02	0.59 ± 0.07	0.25 ± 0.01	0.58 ± 0.05	0.41 ± 0.02
Troglitazone rediscovery	0.48 ± 0.03	0.24 ± 0.02	0.44 ± 0.05	0.24 ± 0.01	0.45 ± 0.11	0.36 ± 0.03
Valsartan smarts	**0.03 ± 0.00**	0.02 ± 0.00	**0.03 ± 0.00**	0.01 ± 0.00	**0.03 ± 0.00**	0.02 ± 0.00
Zaleplon MPO	0.49 ± 0.01	0.36 ± 0.03	0.47 ± 0.01	0.32 ± 0.02	0.53 ± 0.02	0.45 ± 0.01
Total Score	11.96 ± 0.10	7.01 ± 0.13	11.70 ± 0.30	6.59 ± 0.07	12.75 ± 0.22	8.76 ± 0.08
Total Quality	**16.03 ± 0.25**	5.84 ± 0.56	9.96 ± 0.82	3.29 ± 0.29	13.40 ± 0.99	15.12 ± 0.19

Table 4.3: Baseline performance on the GuacaMol benchmark, part II.

Task	MolRL-MGPT	REINFORCE	REINVENT	REINVENT_{MolOpt}	AHC	$\text{ACEGEN}_{Practical}$	ACEGEN_{MolOpt}
Albuterol similarity	0.60 ± 0.02	0.64 ± 0.03	0.60 ± 0.02	0.60 ± 0.02	0.71 ± 0.04	0.82 ± 0.05	**1.00 ± 0.00**
Amlodipine MPO	0.58 ± 0.02	0.61 ± 0.01	0.59 ± 0.01	0.59 ± 0.01	0.63 ± 0.01	0.67 ± 0.02	**0.84 ± 0.06**
Aripiprazole similarity	0.59 ± 0.06	0.58 ± 0.04	0.53 ± 0.02	0.53 ± 0.02	0.64 ± 0.03	0.74 ± 0.07	**1.00 ± 0.00**
C11H24	0.04 ± 0.01	0.58 ± 0.04	0.06 ± 0.02	0.06 ± 0.02	0.06 ± 0.01	0.18 ± 0.08	**0.88 ± 0.05**
C9H10N2O2PF2Cl	0.38 ± 0.02	0.54 ± 0.01	0.48 ± 0.01	0.48 ± 0.01	0.51 ± 0.01	0.48 ± 0.01	**0.81 ± 0.02**
Celecoxib rediscovery	0.65 ± 0.13	0.56 ± 0.03	0.54 ± 0.07	0.54 ± 0.07	0.59 ± 0.03	0.87 ± 0.14	**1.00 ± 0.00**
Deco hop	0.62 ± 0.01	0.62 ± 0.01	0.61 ± 0.01	0.61 ± 0.01	0.63 ± 0.00	0.64 ± 0.01	0.72 ± 0.03
Fexofenadine MPO	0.73 ± 0.01	0.74 ± 0.02	0.74 ± 0.01	0.74 ± 0.01	0.73 ± 0.01	0.74 ± 0.01	**0.87 ± 0.03**
Median molecules 1	0.20 ± 0.01	0.30 ± 0.02	0.25 ± 0.02	0.25 ± 0.02	0.28 ± 0.02	0.28 ± 0.04	**0.39 ± 0.01**
Median molecules 2	0.21 ± 0.01	0.23 ± 0.01	0.23 ± 0.01	0.23 ± 0.01	0.25 ± 0.02	0.25 ± 0.02	**0.36 ± 0.03**
Mestranol similarity	0.54 ± 0.05	0.58 ± 0.01	0.53 ± 0.01	0.53 ± 0.01	0.57 ± 0.02	0.76 ± 0.05	**1.00 ± 0.00**
Osimertinib MPO	0.80 ± 0.01	0.81 ± 0.00	0.80 ± 0.00	0.80 ± 0.00	0.81 ± 0.00	0.80 ± 0.00	**0.87 ± 0.01**
Perindopril MPO	0.48 ± 0.01	0.50 ± 0.00	0.48 ± 0.01	0.48 ± 0.01	0.49 ± 0.01	0.50 ± 0.01	**0.60 ± 0.02**
Ranolazine MPO	0.75 ± 0.01	0.76 ± 0.01	0.75 ± 0.01	0.75 ± 0.01	0.76 ± 0.01	0.76 ± 0.00	0.85 ± 0.01
Scaffold hop	0.52 ± 0.01	0.52 ± 0.01	0.52 ± 0.00	0.52 ± 0.00	0.53 ± 0.01	0.57 ± 0.03	**0.78 ± 0.18**
Sitagliptin MPO	0.28 ± 0.02	0.33 ± 0.03	0.31 ± 0.02	0.31 ± 0.02	0.29 ± 0.02	0.29 ± 0.02	0.48 ± 0.03
Thiothixene rediscovery	0.38 ± 0.02	0.46 ± 0.01	0.42 ± 0.01	0.42 ± 0.01	0.46 ± 0.03	0.61 ± 0.08	**0.98 ± 0.06**
Troglitazone rediscovery	0.36 ± 0.03	0.39 ± 0.05	0.37 ± 0.03	0.37 ± 0.03	0.38 ± 0.03	0.44 ± 0.05	**0.86 ± 0.20**
Valsartan smarts	0.02 ± 0.00	**0.03 ± 0.00**	0.02 ± 0.00	0.02 ± 0.00	0.02 ± 0.00	0.02 ± 0.00	**0.03 ± 0.00**
Zaleplon MPO	0.45 ± 0.01	0.47 ± 0.01	0.47 ± 0.01	0.47 ± 0.01	0.47 ± 0.01	0.47 ± 0.01	**0.55 ± 0.04**
Total Score	9.19 ± 0.16	10.23 ± 0.10	9.29 ± 0.09	9.29 ± 0.09	9.82 ± 0.09	10.88 ± 0.22	**14.85 ± 0.30**
Total Quality	15.00 ± 0.17	15.69 ± 0.19	15.22 ± 0.16	15.22 ± 0.16	15.38 ± 0.20	15.67 ± 0.25	15.72 ± 0.85

5 TTT scaling

This section refers to test-time training scaling, in other words, multiple independent agents trained on the same task, which is generally described in Algorithm 2, further per-step details can be seen in 1.

Algorithm 2: Independent Multi-Agent RL

Input: Agent index set $\mathcal{N} = \{1, \dots, n\}$; policies $\{\pi_{\theta_i}\}_{i \in \mathcal{N}}$; horizon T; batch size B; per-agent budgets $\{M_i\}$; pre-agent replay buffers $\{\mathcal{B}_i\}$, (optional) sigma value σ

Initialize agent policies π_{θ_i} `// Identical policies at initialization`

Initialize counters $N_i \leftarrow 0$ for all $i \in \mathcal{N}$ `// molecules sampled so far by agent i`

(Optional) Initialize frozen prior π_{prior}

while $\exists\, i \in \mathcal{N}$ *s.t.* $N_i < M_i$ **do**

  foreach $i \in \mathcal{N}$ ***with*** $N_i < M_i$ **do**

    $\tau_{\text{on},i}^{B} \leftarrow \text{Rollout}(\pi_{\theta_i}, T, B)$ `// Do rollout`

    $N_i \leftarrow N_i + |\tau_{\text{on},i}^{B_i}|$ `// Update counter`

    $\tau_i^B \leftarrow \tau_{on,i}^{B} \cup \tau_{off,i}$, where $\tau_{off,i} \sim \mathcal{B}_i$ `// Sample from replay buffer`

    $R_i(\tau_i^B) \leftarrow f(\tau_i^B)$ `// Compute rewards`

    $\pi_{\theta_i} \leftarrow \text{Update}_{\Psi}\big(\pi_{\theta_i}, \tau_i^B, R_i\big(\tau_i^B\big), [\pi_{\text{prior}}], [\sigma]\big)$ `// Update policy`

    $\mathcal{B}_i \leftarrow \mathcal{B}_i \cup \text{Top-}k\big(\tau_i^B, R_i\big(\tau_i^B\big)\big)$ `// Update replay buffer`

  end

end

5.1 MolExpL

Table 5.1: Performance on MolExpL benchmark with increasing number of independent REINFORCE agents, each with a budget of 10,000.

Task	1	2	4	8	16	32	64	128
AP	0.56 ± 0.05	0.59 ± 0.03	0.67 ± 0.05	0.64 ± 0.02	0.67 ± 0.03	0.72 ± 0.02	0.72 ± 0.02	**0.75 ± 0.03**
A2A	0.41 ± 0.06	0.42 ± 0.04	0.46 ± 0.05	0.50 ± 0.04	0.55 ± 0.04	0.54 ± 0.04	0.62 ± 0.05	**0.63 ± 0.03**
BACE1	0.22 ± 0.02	0.25 ± 0.02	0.26 ± 0.02	0.29 ± 0.03	0.29 ± 0.02	0.32 ± 0.04	0.34 ± 0.02	**0.37 ± 0.03**
EGFR	0.15 ± 0.02	0.17 ± 0.03	0.17 ± 0.02	0.21 ± 0.02	0.24 ± 0.01	0.24 ± 0.01	0.29 ± 0.02	**0.32 ± 0.02**
Sum	1.34 ± 0.08	1.42 ± 0.06	1.57 ± 0.08	1.64 ± 0.05	1.75 ± 0.06	1.82 ± 0.06	1.97 ± 0.06	**2.08 ± 0.06**

Table 5.2: Performance on MolExpL benchmark with increasing number of independent ACEGEN$_{MolOpt}$ agents, each with a budget of 10,000.

Task	1	2	4	8	16	32	64	128
AP	0.62 ± 0.03	0.64 ± 0.02	0.70 ± 0.06	0.72 ± 0.03	0.70 ± 0.02	0.72 ± 0.01	**0.75 ± 0.05**	**0.75 ± 0.02**
A2A	0.41 ± 0.07	0.51 ± 0.08	0.65 ± 0.08	0.70 ± 0.15	0.76 ± 0.10	0.87 ± 0.13	0.86 ± 0.11	**0.99 ± 0.01**
BACE1	0.31 ± 0.07	0.40 ± 0.03	0.49 ± 0.11	0.53 ± 0.09	0.64 ± 0.07	0.77 ± 0.03	0.82 ± 0.02	**0.90 ± 0.05**
EGFR	0.27 ± 0.06	0.34 ± 0.05	0.46 ± 0.11	0.57 ± 0.07	0.65 ± 0.10	0.77 ± 0.09	0.80 ± 0.05	**0.89 ± 0.04**
Sum	1.62 ± 0.12	1.89 ± 0.10	2.31 ± 0.18	2.51 ± 0.19	2.74 ± 0.15	3.13 ± 0.16	3.24 ± 0.13	**3.52 ± 0.07**

Table 5.3: Performance on MolExpL benchmark with increasing budget for a single ACEGEN$_{MolOpt}$ agent.

Task	10k	20k	40k	80k	160k	320k	640k	1280k
AP	0.62 ± 0.03	0.64 ± 0.02	**0.68 ± 0.05**	0.64 ± 0.03	0.65 ± 0.03	0.65 ± 0.06	0.65 ± 0.04	0.65 ± 0.05
A2A	0.41 ± 0.07	0.45 ± 0.06	0.45 ± 0.06	0.45 ± 0.06	0.45 ± 0.06	0.45 ± 0.06	**0.46 ± 0.06**	**0.46 ± 0.06**
BACE1	0.31 ± 0.07	0.30 ± 0.07	**0.35 ± 0.03**	0.32 ± 0.02	0.32 ± 0.06	0.32 ± 0.07	0.33 ± 0.03	0.34 ± 0.06
EGFR	0.27 ± 0.06	0.25 ± 0.04	**0.28 ± 0.05**	0.27 ± 0.03	0.26 ± 0.03	0.25 ± 0.02	0.24 ± 0.04	0.25 ± 0.06
Sum	1.62 ± 0.12	1.64 ± 0.11	**1.76 ± 0.10**	1.68 ± 0.08	1.68 ± 0.09	1.67 ± 0.11	1.67 ± 0.09	1.70 ± 0.12

Table 5.4: Performance on MolExpL benchmark with increasing budget for a single ACEGEN$_{MolOpt}$ agent with a RND exploration bonus.

Task	10k	20k	40k	80k	160k	320k	640k	1280k
AP	0.65 ± 0.03	0.66 ± 0.03	0.65 ± 0.04	0.65 ± 0.01	0.65 ± 0.02	0.66 ± 0.04	0.65 ± 0.01	**0.67 ± 0.03**
A2A	0.43 ± 0.03	0.45 ± 0.03	**0.47 ± 0.06**	**0.47 ± 0.06**	**0.47 ± 0.06**	**0.47 ± 0.06**	**0.47 ± 0.06**	**0.47 ± 0.06**
BACE1	0.23 ± 0.01	**0.38 ± 0.05**	0.35 ± 0.06	0.32 ± 0.03	0.34 ± 0.03	0.37 ± 0.04	**0.38 ± 0.05**	0.37 ± 0.02
EGFR	0.18 ± 0.02	0.26 ± 0.04	**0.32 ± 0.04**	**0.32 ± 0.07**	0.31 ± 0.04	0.30 ± 0.03	0.30 ± 0.03	0.29 ± 0.06
Sum	1.50 ± 0.05	1.75 ± 0.08	1.79 ± 0.10	1.76 ± 0.09	1.76 ± 0.08	1.80 ± 0.09	**1.81 ± 0.09**	1.80 ± 0.09

Table 5.5: Performance on MolExpL benchmark with increasing budget for a single ACEGEN$_{MolOpt}$ agent with DF penalization.

Task	10k	20k	40k	80k	160k	320k	640k	1280k
AP	**0.66 ± 0.02**	**0.66 ± 0.04**	0.65 ± 0.03	0.64 ± 0.02	0.65 ± 0.05	0.64 ± 0.02	0.65 ± 0.05	0.65 ± 0.02
A2A	**0.50 ± 0.04**	0.45 ± 0.04	**0.50 ± 0.04**	**0.50 ± 0.04**	**0.50 ± 0.04**	**0.50 ± 0.04**	**0.50 ± 0.04**	0.49 ± 0.02
BACE1	0.29 ± 0.05	0.38 ± 0.08	0.34 ± 0.09	0.35 ± 0.04	0.33 ± 0.03	0.32 ± 0.08	**0.40 ± 0.08**	0.37 ± 0.06
EGFR	0.23 ± 0.06	0.27 ± 0.03	0.27 ± 0.06	0.24 ± 0.03	0.25 ± 0.04	**0.32 ± 0.04**	0.29 ± 0.07	0.28 ± 0.05
Sum	1.67 ± 0.09	1.77 ± 0.10	1.76 ± 0.12	1.72 ± 0.06	1.72 ± 0.08	1.78 ± 0.10	**1.84 ± 0.12**	1.79 ± 0.08

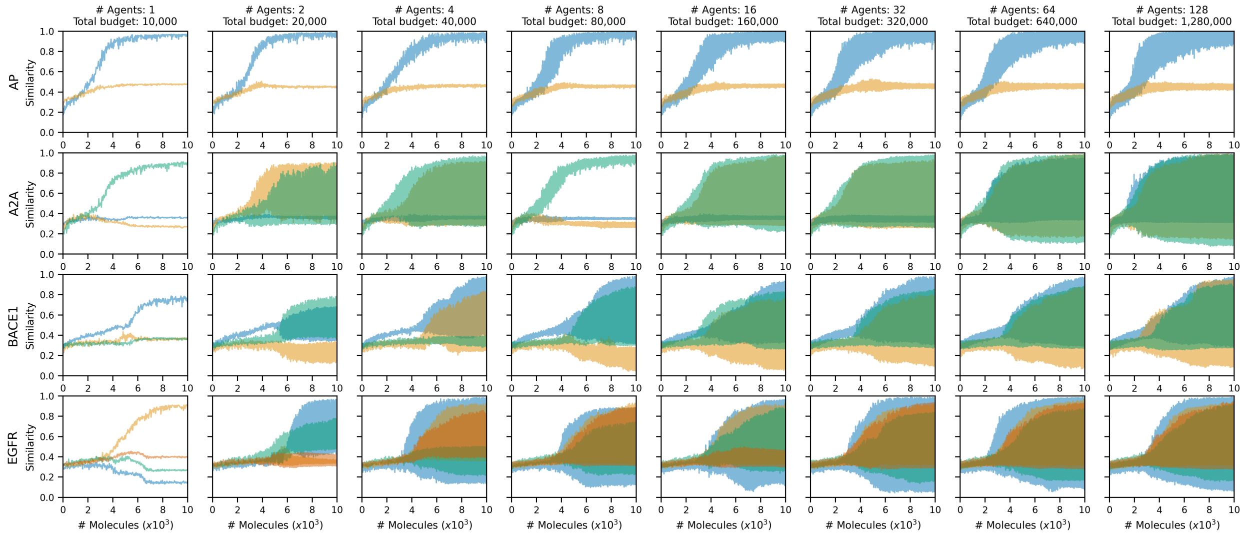

Figure 5.1: Multiple independent ACEGEN$_{MolOpt}$ agents on the MolExpL benchmark tasks, single replicate. Each line represents average similarity to one of the tasks target molecules, and for clarity the standard deviation is not plotted due to already high variance across agents. As the number of agents increases (increasing the total budget) stochasticity increases, resulting in divergence in agent behavior. Interestingly, even with 128 independent agents all seem to choose maximization towards one AP target molecule over the other.

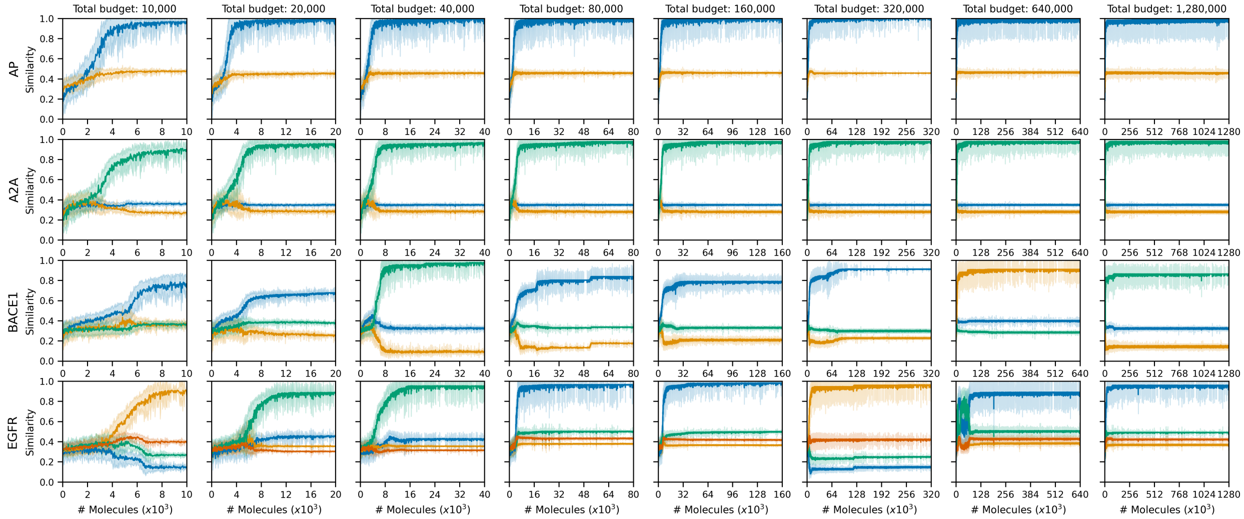

Figure 5.2: Single ACEGEN$_{MolOpt}$ agent on the MolExpL benchmark tasks, single replicate. Each line represents average similarity to one of the tasks target molecules. In general, a single agent optimizes for similarity to one target molecule and doesn't change throughout training. One notable exception exists for EGFR with a budget of 640,000, where the agent seems to switch molecular target (in this case from green to blue).

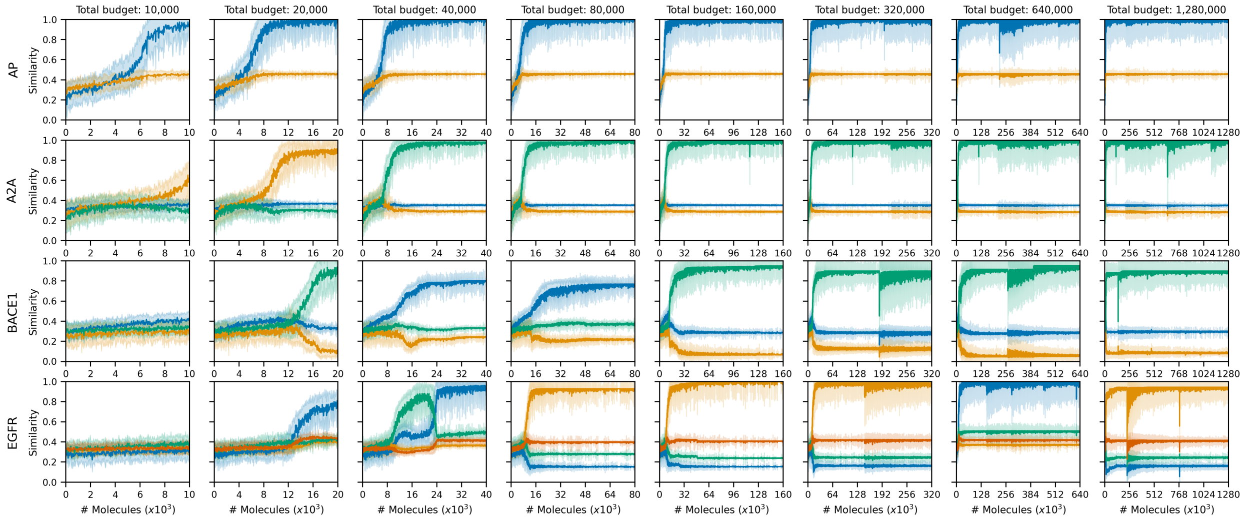

Figure 5.3: Single ACEGEN$_{MolOpt}$ agent with RND exploration bonus on the MolExpL benchmark tasks, single replicate. Each line represents average similarity to one of the tasks target molecules. In general, a single agent optimizes for similarity to one target molecule and doesn't change throughout training, even with exploration bonuses. Note that average score can drop from a budget of 320,000 onward, but doesn't resulted in a switching of target molecule.

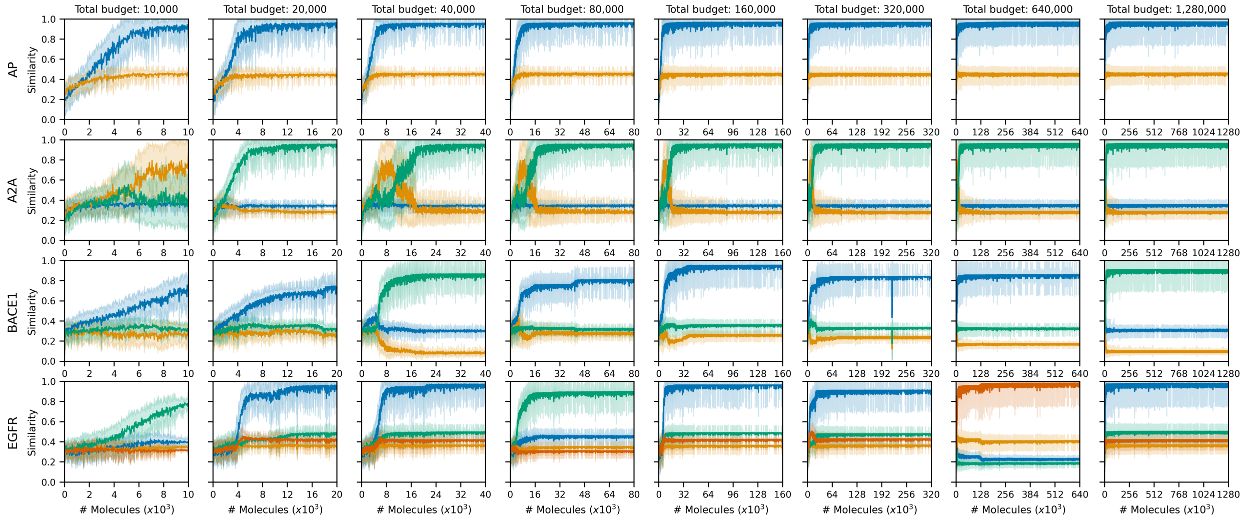

Figure 5.4: Single ACEGEN$_{MolOpt}$ agent with DF penalization on the MolExpL benchmark tasks, single replicate. Each line represents average similarity to one of the tasks target molecules.

5.2 MolExp

Table 5.6: Performance on MolExp benchmark with increasing number of independent ACEGEN$_{MolOpt}$ agents, each with a budget of 10,000.

Task	1	2	4	8	16	32	64	128
AP	0.42 ± 0.04	0.43 ± 0.03	0.50 ± 0.04	0.50 ± 0.02	0.48 ± 0.03	0.51 ± 0.02	0.54 ± 0.01	**0.60 ± 0.09**
A2A	0.13 ± 0.05	0.13 ± 0.02	0.15 ± 0.03	0.16 ± 0.02	0.28 ± 0.15	0.30 ± 0.12	0.44 ± 0.12	**0.61 ± 0.23**
BACE1	0.06 ± 0.01	0.07 ± 0.01	0.09 ± 0.01	0.10 ± 0.02	0.11 ± 0.02	0.12 ± 0.02	0.15 ± 0.04	**0.18 ± 0.04**
EGFR	0.06 ± 0.01	0.08 ± 0.04	0.12 ± 0.03	0.18 ± 0.02	0.21 ± 0.06	0.24 ± 0.04	0.31 ± 0.05	**0.36 ± 0.03**
Sum	0.66 ± 0.07	0.71 ± 0.05	0.86 ± 0.06	0.94 ± 0.04	1.08 ± 0.16	1.17 ± 0.13	1.44 ± 0.14	**1.75 ± 0.25**

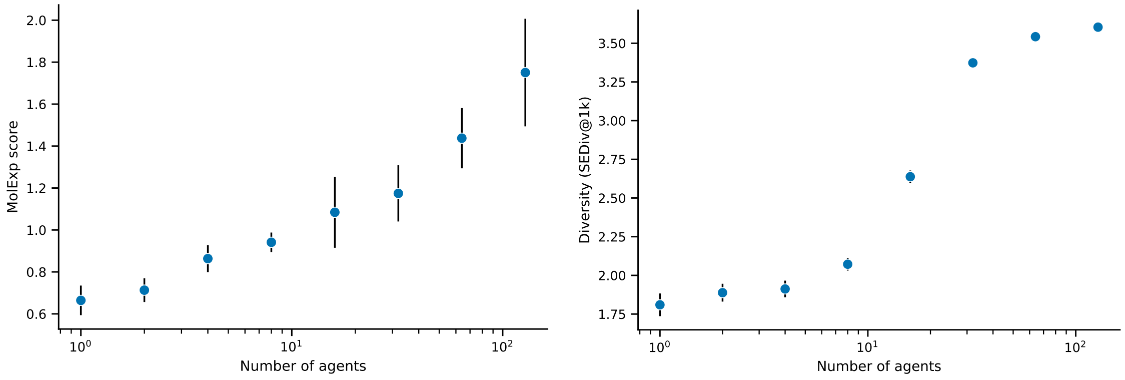

(a) Scaling RL agents

(b) Molecular diversity

Figure 5.5: Performance on the MolExp benchmark with scaling. (a) Scaling the number of independent ACEGEN$_{MolOpt}$, each with a budget of 10,000. (b) The diversity of sampled compounds as measured by sphere exclusion diversity.

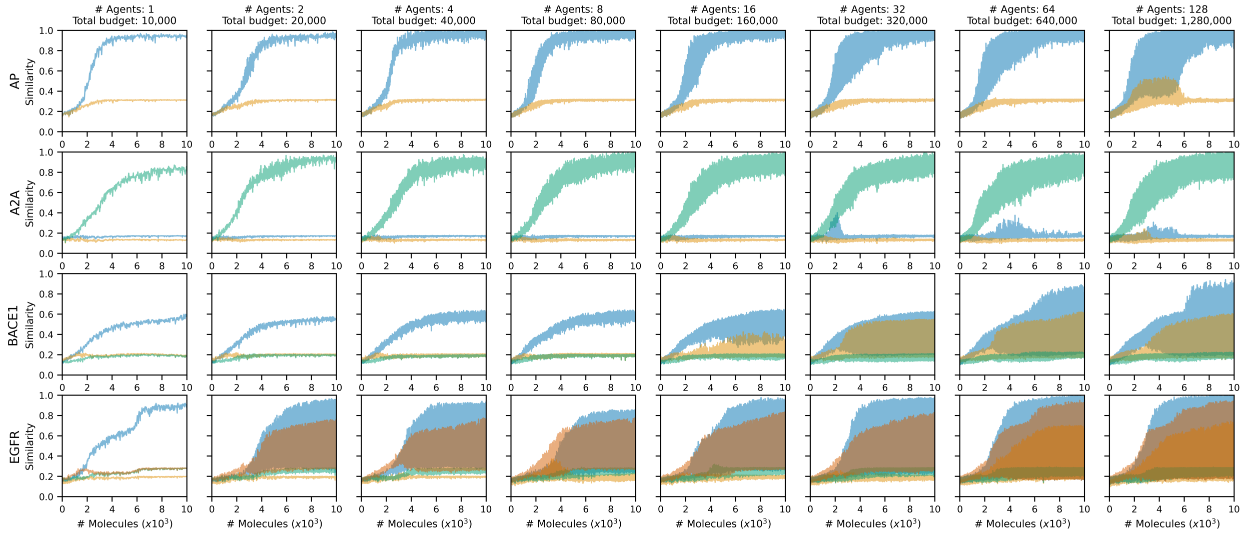

Figure 5.6: Multiple independent ACEGEN$_{MolOpt}$ agents on the MolExp benchmark tasks, single replicate. Each line represents average similarity to one of the tasks target molecules, and for clarity the standard deviation is not plotted due to already high variance across agents.

5.3 GuacaMol

Table 5.7: Performance on GuacaMol benchmark with increasing number of independent ACEGEN$_{MolOpt}$ agents, each with a budget of 10,000.

Task	1	2	4	8	16	32
Albuterol similarity	**1.00 ± 0.00**	**1.00 ± 0.00**	**1.00 ± 0.00**	**1.00 ± 0.00**	**1.00 ± 0.00**	**1.00 ± 0.00**
Amlodipine MPO	0.84 ± 0.06	0.86 ± 0.03	0.89 ± 0.00	0.89 ± 0.00	**0.90 ± 0.00**	**0.90 ± 0.00**
Aripiprazole similarity	**1.00 ± 0.00**	**1.00 ± 0.00**	**1.00 ± 0.00**	**1.00 ± 0.00**	**1.00 ± 0.00**	**1.00 ± 0.00**
C11H24	0.88 ± 0.05	0.92 ± 0.04	0.97 ± 0.02	0.99 ± 0.00	0.99 ± 0.00	**1.00 ± 0.00**
C9H10N2O2PF2Cl	0.81 ± 0.02	0.83 ± 0.03	0.85 ± 0.01	0.88 ± 0.00	0.88 ± 0.00	**0.89 ± 0.01**
Celecoxib rediscovery	**1.00 ± 0.00**	**1.00 ± 0.00**	**1.00 ± 0.00**	**1.00 ± 0.00**	**1.00 ± 0.00**	**1.00 ± 0.00**
Deco hop	0.72 ± 0.03	0.73 ± 0.02	0.73 ± 0.02	0.73 ± 0.01	**0.75 ± 0.00**	**0.75 ± 0.00**
Fexofenadine MPO	0.87 ± 0.03	0.90 ± 0.02	0.89 ± 0.01	0.91 ± 0.01	0.92 ± 0.01	**0.93 ± 0.01**
Median molecules 1	0.39 ± 0.01	0.41 ± 0.03	0.42 ± 0.01	**0.44 ± 0.01**	**0.44 ± 0.01**	**0.44 ± 0.00**
Median molecules 2	0.36 ± 0.03	0.37 ± 0.02	0.39 ± 0.02	0.40 ± 0.01	**0.41 ± 0.01**	0.40 ± 0.00
Mestranol similarity	**1.00 ± 0.00**	**1.00 ± 0.01**	**1.00 ± 0.00**	**1.00 ± 0.00**	**1.00 ± 0.00**	**1.00 ± 0.00**
Osimertinib MPO	0.87 ± 0.01	0.88 ± 0.01	0.88 ± 0.01	0.89 ± 0.01	0.89 ± 0.00	**0.90 ± 0.01**
Perindopril MPO	0.60 ± 0.02	0.65 ± 0.03	0.65 ± 0.05	0.71 ± 0.05	0.72 ± 0.04	**0.76 ± 0.01**
Ranolazine MPO	0.85 ± 0.01	0.85 ± 0.01	0.85 ± 0.00	0.85 ± 0.01	**0.87 ± 0.01**	**0.87 ± 0.01**
Scaffold hop	0.78 ± 0.18	0.86 ± 0.13	0.79 ± 0.18	0.94 ± 0.06	**0.99 ± 0.00**	**0.99 ± 0.01**
Sitagliptin MPO	0.48 ± 0.03	0.47 ± 0.04	0.52 ± 0.07	0.56 ± 0.04	0.58 ± 0.03	**0.61 ± 0.06**
Thiothixene rediscovery	0.98 ± 0.06	**1.00 ± 0.00**	**1.00 ± 0.00**	**1.00 ± 0.00**	**1.00 ± 0.00**	**1.00 ± 0.00**
Troglitazone rediscovery	0.86 ± 0.20	0.87 ± 0.17	**1.00 ± 0.00**	**1.00 ± 0.00**	**1.00 ± 0.00**	**1.00 ± 0.00**
Valsartan smarts	**0.03 ± 0.00**	**0.03 ± 0.00**	**0.03 ± 0.00**	**0.03 ± 0.00**	**0.03 ± 0.00**	**0.03 ± 0.00**
Zaleplon MPO	0.55 ± 0.04	0.55 ± 0.00	0.58 ± 0.01	0.58 ± 0.02	0.61 ± 0.03	**0.62 ± 0.03**
GuacaMol Score	14.85 ± 0.30	15.17 ± 0.23	15.47 ± 0.20	15.79 ± 0.10	15.98 ± 0.06	**16.10 ± 0.07**
GuacaMol Quality	**15.72 ± 0.85**	15.05 ± 0.52	15.27 ± 0.53	14.53 ± 0.50	14.25 ± 0.44	13.99 ± 0.45

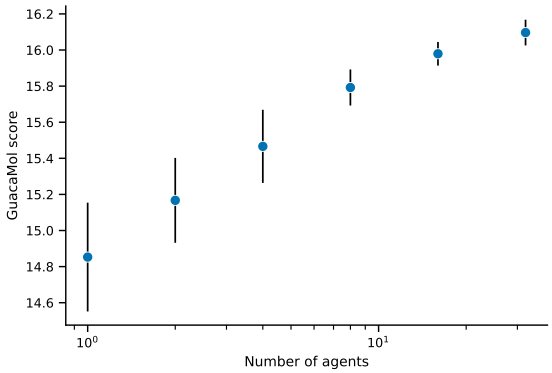

(a) Scaling RL agents

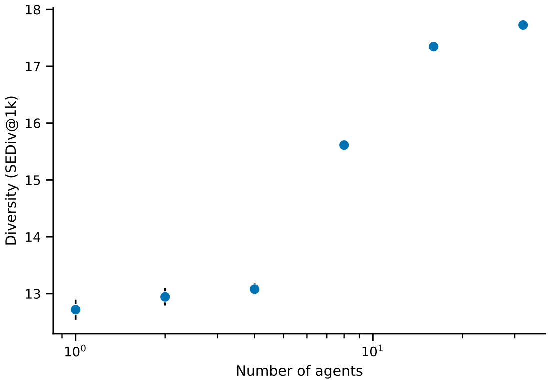

(b) Molecular diversity

Figure 5.7: Performance on the GuacaMol benchmark with scaling. (a) Scaling the number of independent ACEGEN_{MolOpt}, each with a budget of 10,000. (b) The diversity of sampled compounds as measured by sphere exclusion diversity.

6 Cooperative strategies

We employed the following cooperative strategies using $\langle \mathcal{S}, \mathcal{A}^i, R, P^i \rangle$ for $i \in \mathcal{N}$ where the states S and R may be shared across agents in a cooperative manner. For brevity, we denote the probability of a full sequence generated as $\pi_i(\tau)$. These cooperative strategies are adaptations to the general algorithm outlined in Algorithm 2.

Purge Purging each agent's replay buffer $\mathcal{B}_i$ to ensure no molecule exists in more than one replay buffer, resulting in a unique replay buffer for each agent.

$$\forall \tau \in \mathcal{B}_i, \quad \tau \notin \mathcal{B}_j \quad \text{for all } j \neq i. \tag{6.1}$$

Shared One shared replay buffer $\mathcal{B}$ is used by all agents as a method of communicating high-rewarding areas of chemical space by on-policy augmentation. We anticipate this to result in convergence of agents to the same areas of chemical space and hence act as a negative control.

Shared with bonus The same as **Shared**, however, an additional intrinsic reward $R(\tau)_{Nov}$ was applied as a novelty bonus for molecules not already contained within the replay buffer $\mathcal{B}$. This reward shaping was applied before the ACEGEN reward shaping mechanisms outlined in the main body of text.

$$R(\tau)_{reshaped} = \frac{R(\tau) + R(\tau)_{Nov}}{2} \tag{6.2}$$

Noise Gaussian noise was applied to each agent's parameters once at initialization. Such as to perturb each agent's starting policy to encourage learning divergence. The effect of λ was tested on the validity of generated SMILES strings Figure 6.1. In cooperative experiments, we applied $\lambda_{Noise} = 0.01$ noise to all GRU hidden layers and the final linear layer.

$$\theta'_{step=0} = \theta_{step=0} + \lambda \cdot \mathcal{N}(0,1) \tag{6.3}$$

RND Random network distillation (RND) was applied to provide an exploration bonus to each agent to universally novel molecules. This bonus is calculated as the squared error between a target network $f(x)$ and predictor network $\hat{f}(x)$ mapping of x. The predictor network is trained iteratively on all visited states $s \in \mathcal{S}$.

$$R(\tau)' = R(\tau) + \lambda \cdot ||\hat{f}(x;\theta) - f(x)||^2 \tag{6.4}$$

The networks used in our implementation employ the same architecture as the CLM agents. The predictor network is trained to minimize the log-likelihood of SMILES sequences. The bonus is computed as the difference in policies between SMILES sequences measured by the summed log-likelihoods $\sum_{t=1}^{T} \log P_\theta(x_t|x_{<t})$.

ENT$_\mathcal{S}$ An additional loss term for the k-th agent was added to minimize the entropy across on-policy final states of agents $\mathcal{S}^{\{1,\ldots,K\}\subseteq\mathcal{N}}$, to encourage the preferential sampling of states sampled by the k-th agent and discourage states sampled by other agents. To measure the likelihood of an agent sampling a state we used perplexity ($\mathcal{P}$).

$$\mathcal{P}(\tau) = e^{-\frac{1}{T}\sum_{t=1}^{T} \log \pi(a_t|s_t)} \tag{6.5}$$

The perplexities given the k-th agent policy π_k were summed over on-policy states of agents $\mathcal{S}^{\{1,\ldots,K\}\subseteq\mathcal{N}}$ resulting in a state-conditioned policy uncertainty value for each set of agent i states $\mathcal{P}_{\text{sum}}(\pi_k, \mathcal{S}^i)$. The negative of this vector (so that smaller numbers represent more uncertainty) was softmax normalized resulting in a pseudo-probability $\tilde{P}(\mathcal{S}^i|\pi_k)$ of agent k sampling states derived from different agents.

$$\mathcal{L}^k_{ENT_\mathcal{S}} = \lambda \cdot \sum_{i=1}^{k} \tilde{P}(\mathcal{S}^i|\pi_k) \log \tilde{P}(\mathcal{S}^i|\pi_k) \tag{6.6}$$

CE$_\mathcal{S}$ A natural extension from **ENT$_\mathcal{S}$** is the addition of a cross-entropy loss term. The same steps from **ENT$_\mathcal{S}$** were followed to result in a pseudo-probability $\tilde{P}(\mathcal{S}^i|\pi_k)$ of agent k sampling states derived from different agents. Then cross-entropy was applied to explicitly indicate that the k-th agent should assign a higher pseudo-probability to sampling states it has collected itself $\mathcal{S}^k$ than states collected by other agents $\mathcal{S}^i$.

$$\mathcal{L}^k_{CE_\mathcal{S}} = -\lambda \cdot \log \tilde{P}(\mathcal{S}^k|\pi_k) \tag{6.7}$$

DIFF$_\mathcal{S}$ An additional loss term for the k-th agent to explicitly maximize the difference between uncertainty of states collected by previous agents relative to those collected by itself. Similar to **ENT$_\mathcal{S}$** the summed perplexity $\mathcal{P}_{\text{sum}}(\pi_k, \mathcal{S}^i)$ for collected states given the k-th agent policy was calculated.

$$\mathcal{L}^k_{DIFF_\mathcal{S}} = -\lambda \cdot |\frac{\sum_{i=1}^{k-1} \mathcal{P}_{\text{sum}}(\pi_k, \mathcal{S}^i)}{k-1} - \mathcal{P}_{\text{sum}}(\pi_k, \mathcal{S}^k)| \tag{6.8}$$

DIFF$_\mathcal{N}$ As initially proposed by Hu et al.[S4], an additional loss penalty was added which seeks to maximize the difference in log-probability for on-policy states $\mathcal{S}^k$ between the k-th agent and previous agents, scaled by the reward $R(\tau)$.

$$\mathcal{L}^k_{DIFF_\mathcal{N}} = -\lambda \cdot \sum_{i=1}^{k-1} R(\tau) \cdot |\log \pi_k(\tau) - \log \pi_i(\tau)| \tag{6.9}$$

DvD Taking inspiration from Parker-Holder et al.[S7], we implemented Diversity via Deter-

minant to enforce divergent behavior of agent policies. A random selection of 100 on-policy states were sampled and used to form an embedding that encoded policy behavior. This embedding was formed by concatenating the transition probabilities for possible actions $P(s^i_{t+1}|a^i_t, s_t)$ for a given agent policy π_i for each sampled state s. Let this form the agent embedding $\mathbf{e_i}$ for each agent i which were then used to compute pairwise similarities via a radial basis function (RBF) kernel:

$$K_{ij} = \exp\left(-\frac{\|\mathbf{e}_i - \mathbf{e}_j\|^2}{2\sigma^2}\right), \tag{6.10}$$

where K_{ij} is the kernel value between agents i and j, $\|\mathbf{e}_i - \mathbf{e}_j\|^2$ is the squared Euclidean distance, and σ is a kernel bandwidth parameter.

The determinant of the kernel matrix K, denoted as $\det(K)$, was used as a measure of policy divergence which quantifies diversity, with higher values indicating greater divergence in agent policies and hence, was used as a penalty term:

$$\mathcal{L}^i_{\text{DvD}} = -\det(K), \tag{6.11}$$

POPNORM Population normalization was used as a strategy to encourage divergent behavior from previous agents by adding a penalty term of the average population return up to the current k-th agent.

$$\mathcal{L}^k_{\text{POPNORM}} = -\frac{1}{k-1}\sum_{i=1}^{k-1} \pi_i(\tau) \cdot R(\tau) \tag{6.12}$$

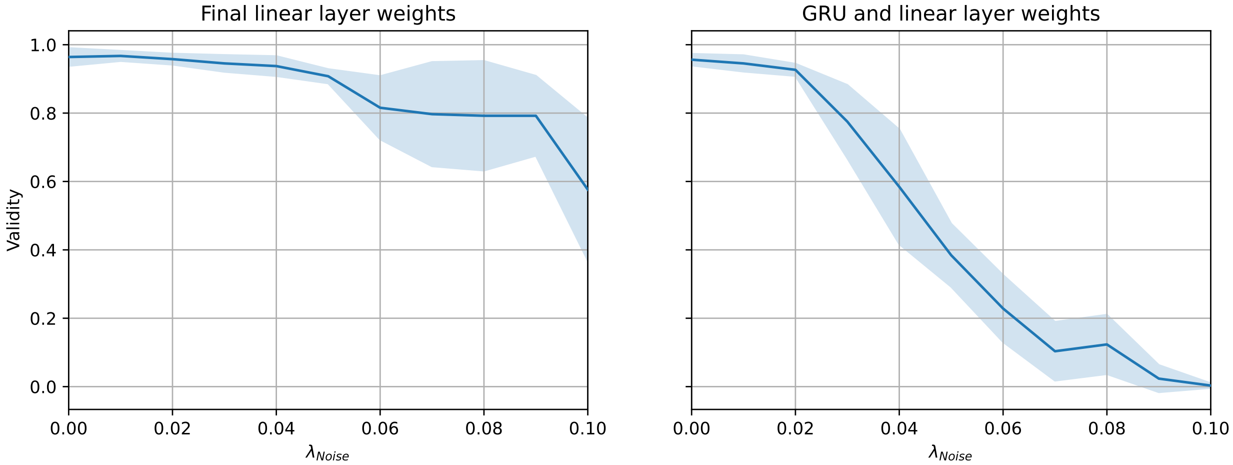

Figure 6.1: Application of noise with varying coefficients λ_{Noise} to either the final linear layer weights or all GRU hidden layers and the final linear layer weights. Increasing amounts of noise decrease the validity of molecules generated.

7 Cooperative RL

7.1 MolExpL

Table 7.1: Performance on MolExpL benchmark of different 4-agent cooperative strategies, part I.

Task	Independent	Noise	Shared	Shared w. bonus	Purge	RND	DvD	POPNORM	MolRL-MGPT
AP	**0.70 ± 0.06**	0.66 ± 0.02	0.68 ± 0.04	0.64 ± 0.01	0.65 ± 0.02	0.68 ± 0.02	0.69 ± 0.04	0.67 ± 0.03	0.54 ± 0.02
A2A	0.65 ± 0.08	0.64 ± 0.18	0.51 ± 0.12	0.55 ± 0.11	0.63 ± 0.05	0.66 ± 0.07	0.68 ± 0.10	0.68 ± 0.08	0.52 ± 0.05
BACE1	0.49 ± 0.11	0.53 ± 0.10	0.39 ± 0.03	0.37 ± 0.03	0.42 ± 0.13	0.35 ± 0.06	0.40 ± 0.05	0.53 ± 0.08	0.31 ± 0.03
EGFR	0.46 ± 0.11	0.44 ± 0.06	0.29 ± 0.06	0.29 ± 0.03	0.45 ± 0.06	0.41 ± 0.06	0.43 ± 0.05	0.48 ± 0.05	0.23 ± 0.04
Sum	2.31 ± 0.18	2.27 ± 0.21	1.86 ± 0.14	1.86 ± 0.12	2.16 ± 0.15	2.10 ± 0.11	2.19 ± 0.13	2.35 ± 0.13	1.59 ± 0.07

Table 7.2: Performance on MolExpL benchmark of different 4-agent cooperative strategies, part II.

Task	ENT_S-0.001	ENT_S-0.01	ENT_S-0.1	ENT_S-1	CE_S-0.001	CE_S-0.01	CE_S-0.1	CE_S-1
AP	0.66 ± 0.03	0.67 ± 0.03	0.65 ± 0.02	0.68 ± 0.02	0.69 ± 0.03	0.68 ± 0.01	0.68 ± 0.04	0.68 ± 0.04
A2A	0.67 ± 0.20	0.72 ± 0.12	0.65 ± 0.12	0.60 ± 0.08	0.65 ± 0.18	0.62 ± 0.07	0.73 ± 0.13	0.70 ± 0.09
BACE1	**0.59 ± 0.08**	0.51 ± 0.17	0.50 ± 0.09	0.41 ± 0.05	0.46 ± 0.15	0.53 ± 0.08	0.43 ± 0.05	0.36 ± 0.07
EGFR	0.47 ± 0.05	0.44 ± 0.10	0.47 ± 0.09	0.38 ± 0.07	0.46 ± 0.15	0.44 ± 0.10	0.47 ± 0.08	0.39 ± 0.11
Sum	**2.39 ± 0.22**	2.33 ± 0.23	2.28 ± 0.18	2.07 ± 0.12	2.25 ± 0.28	2.27 ± 0.14	2.31 ± 0.16	2.13 ± 0.16

Table 7.3: Performance on MolExpL benchmark of different 4-agent cooperative strategies, part III.

Task	DIFF_S-0.001	DIFF_S-0.01	DIFF_S-0.1	DIFF_S-1	DIFF_N-0.001	DIFF_N-0.01	DIFF_N-0.1	DIFF_N-1
AP	0.68 ± 0.02	0.69 ± 0.02	0.66 ± 0.03	0.63 ± 0.03	0.67 ± 0.03	0.68 ± 0.03	0.66 ± 0.02	0.68 ± 0.04
A2A	0.57 ± 0.07	0.61 ± 0.08	0.54 ± 0.06	0.46 ± 0.04	0.68 ± 0.14	0.64 ± 0.04	0.63 ± 0.12	**0.74 ± 0.07**
BACE1	0.45 ± 0.06	0.46 ± 0.06	0.35 ± 0.04	0.31 ± 0.06	0.48 ± 0.11	0.43 ± 0.13	0.47 ± 0.07	0.47 ± 0.13
EGFR	0.45 ± 0.10	0.44 ± 0.05	0.36 ± 0.10	0.26 ± 0.05	0.50 ± 0.04	**0.53 ± 0.03**	0.45 ± 0.05	0.50 ± 0.10
Sum	2.14 ± 0.13	2.20 ± 0.12	1.92 ± 0.13	1.66 ± 0.10	2.33 ± 0.19	2.27 ± 0.14	2.21 ± 0.15	**2.39 ± 0.18**

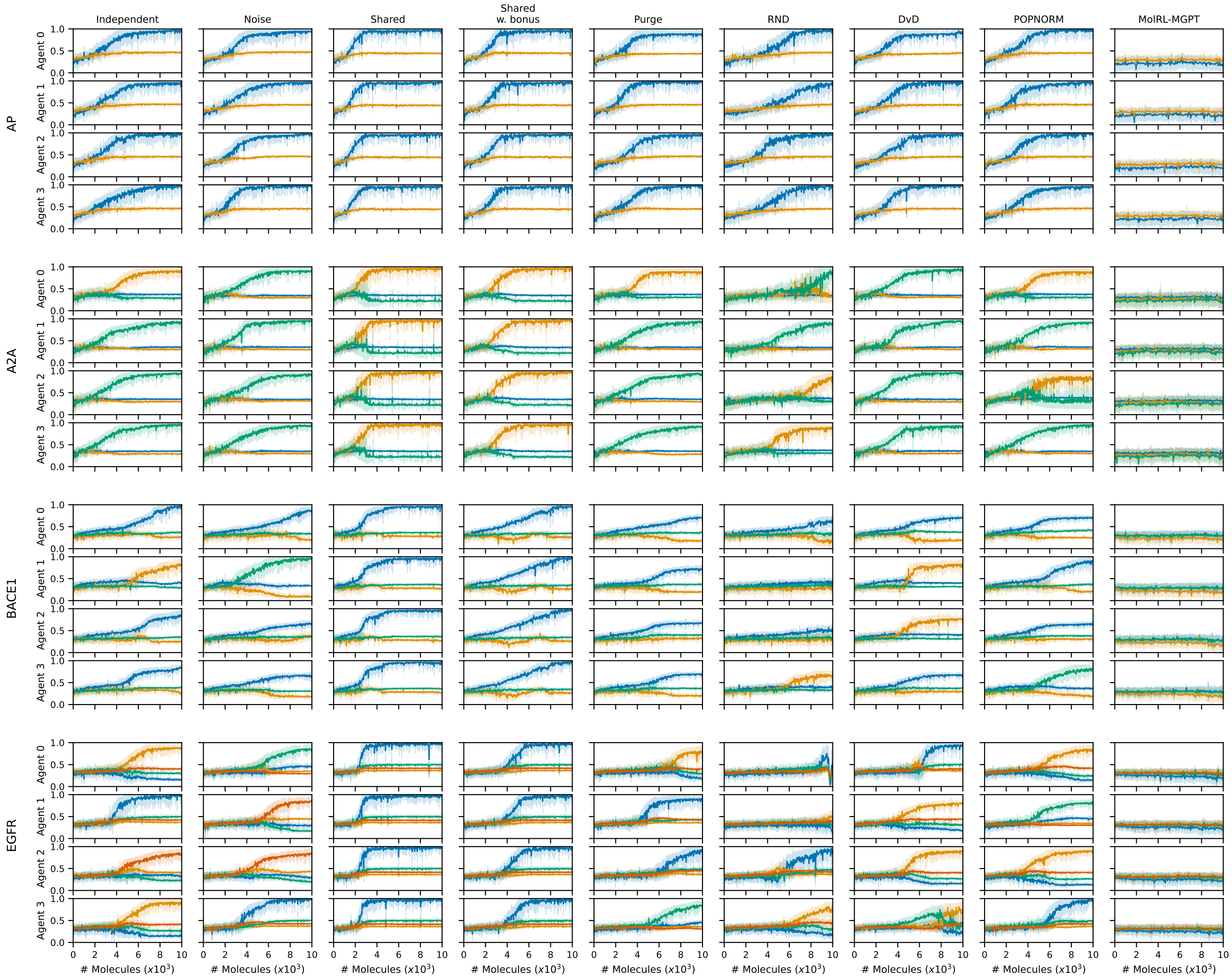

Figure 7.1: Cooperative strategies using 4-agents on the MolExpL benchmark tasks, single replicate, part I. Each line represents average similarity to one of the tasks target molecules. Training of each agent is plotted. Perfect cooperation is each agent learning to rediscover a different target molecule. Most cooperative strategies result in no additional divergent behavior or in some cases, slower learning.

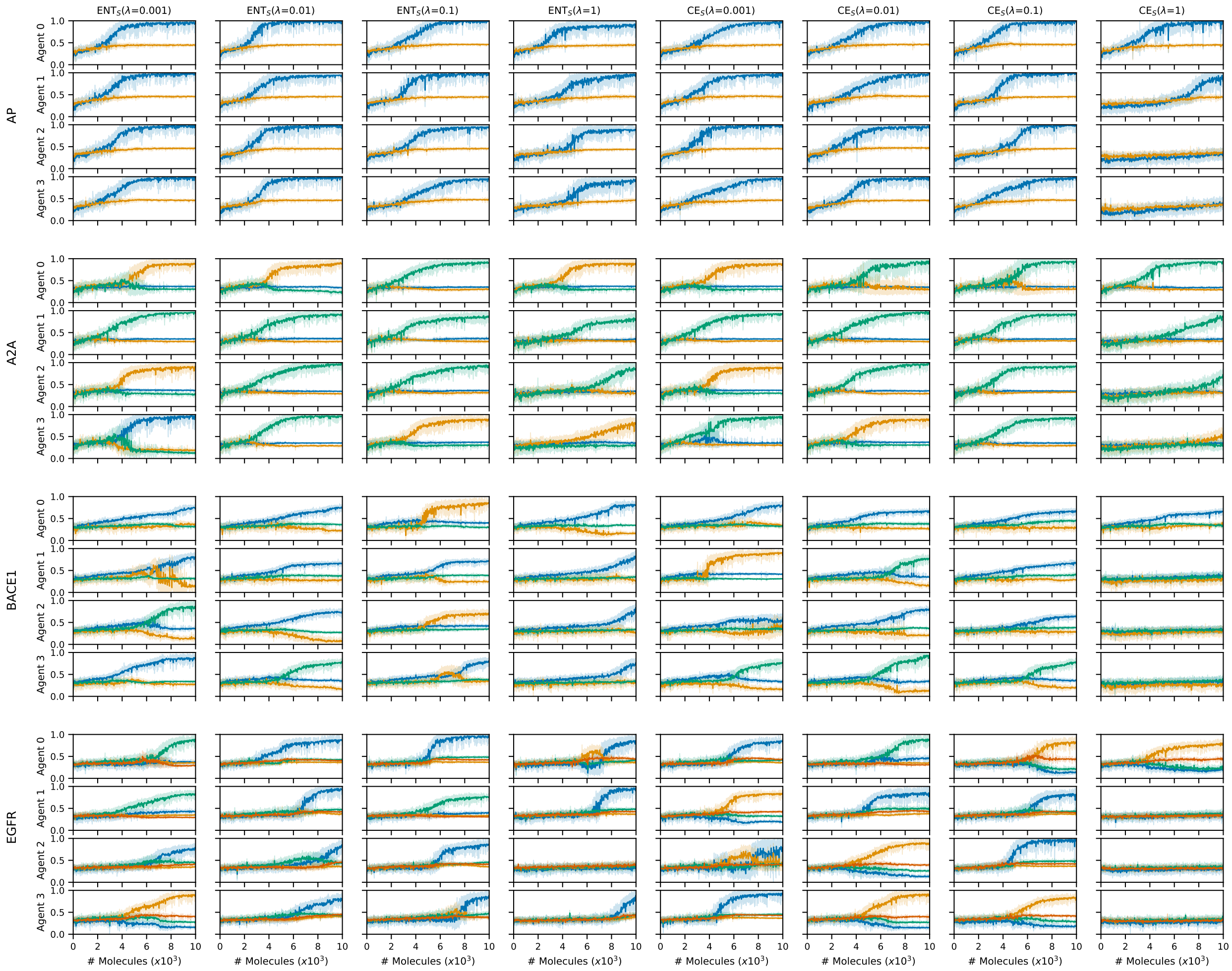

Figure 7.2: Cooperative strategies using 4-agents on the MolExpL benchmark tasks, single replicate, part II. Each line represents average similarity to one of the tasks target molecules. Training of each agent is plotted. Perfect cooperation is each agent learning to rediscover a different target molecule. Most cooperative strategies result in no additional divergent behavior or in some cases, slower learning.

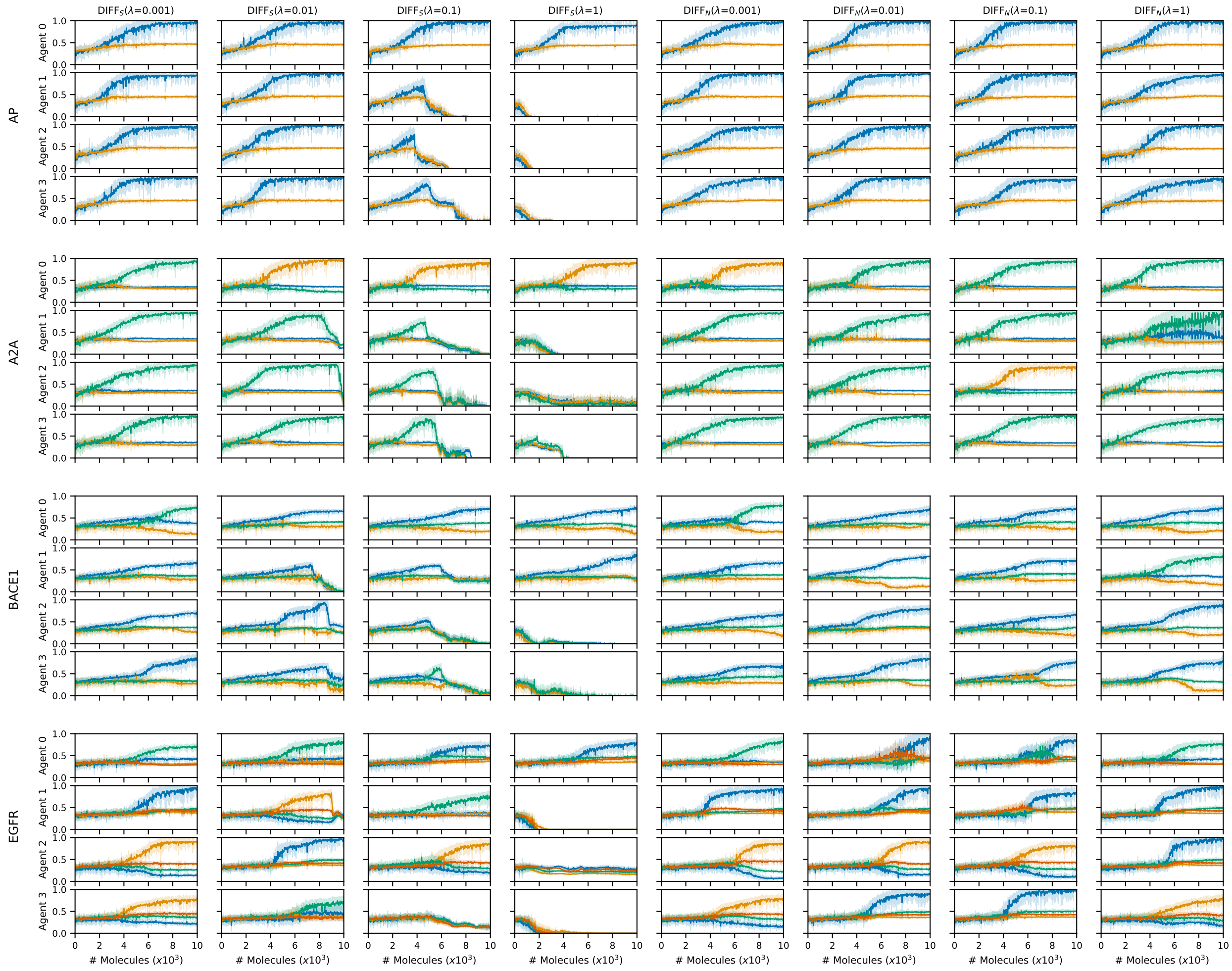

Figure 7.3: Cooperative strategies using 4-agents on the MolExpL benchmark tasks, single replicate, part III. Each line represents average similarity to one of the tasks target molecules. Training of each agent is plotted. Perfect cooperation is each agent learning to rediscover a different target molecule. Most cooperative strategies result in no additional divergent behavior or in some cases, slower learning.

7.2 MolExp

Table 7.4: Performance on MolExp benchmark of different 4-agent cooperative strategies, part I.

Task	Independent	Noise	Shared	Shared w. bonus	Purge	RND	DvD	POPNORM	MolRL-MGPT
AP	0.50 ± 0.04	0.47 ± 0.04	0.43 ± 0.02	0.44 ± 0.02	0.44 ± 0.01	0.47 ± 0.04	0.48 ± 0.06	0.46 ± 0.03	0.31 ± 0.06
A2A	0.15 ± 0.03	0.25 ± 0.13	0.15 ± 0.04	0.17 ± 0.03	0.17 ± 0.05	0.17 ± 0.04	0.20 ± 0.12	0.17 ± 0.06	0.12 ± 0.02
BACE1	0.09 ± 0.01	0.09 ± 0.01	0.07 ± 0.01	0.07 ± 0.01	0.08 ± 0.01	0.08 ± 0.01	0.07 ± 0.00	0.08 ± 0.01	**0.15 ± 0.04**
EGFR	0.12 ± 0.03	0.11 ± 0.04	0.07 ± 0.02	0.06 ± 0.01	0.12 ± 0.04	0.08 ± 0.01	0.11 ± 0.02	0.13 ± 0.10	0.05 ± 0.01
Sum	**1.30 ± 0.07**	0.91 ± 0.14	0.73 ± 0.05	0.74 ± 0.04	0.81 ± 0.07	0.80 ± 0.06	0.86 ± 0.13	0.85 ± 0.12	0.62 ± 0.07

Table 7.5: Performance on MolExp benchmark of different 4-agent cooperative strategies, part II.

Task	ENT$_S$-0.001	ENT$_S$-0.01	ENT$_S$-0.1	ENT$_S$-1	CE$_S$-0.001	CE$_S$-0.01	CE$_S$-0.1	CE$_S$-1
AP	0.45 ± 0.04	0.45 ± 0.03	**0.54 ± 0.07**	0.47 ± 0.04	0.51 ± 0.04	0.49 ± 0.06	**0.54 ± 0.09**	0.48 ± 0.05
A2A	0.15 ± 0.02	0.25 ± 0.12	0.23 ± 0.16	0.25 ± 0.13	0.22 ± 0.11	0.15 ± 0.03	**0.42 ± 0.07**	0.27 ± 0.12
BACE1	0.11 ± 0.06	0.08 ± 0.00	0.09 ± 0.02	0.09 ± 0.01	0.08 ± 0.02	0.08 ± 0.01	0.11 ± 0.03	0.09 ± 0.01
EGFR	0.13 ± 0.05	0.13 ± 0.04	0.12 ± 0.05	0.10 ± 0.03	0.10 ± 0.03	0.12 ± 0.04	0.14 ± 0.04	0.08 ± 0.04
Sum	0.83 ± 0.08	0.91 ± 0.12	0.98 ± 0.18	0.91 ± 0.14	0.91 ± 0.13	0.84 ± 0.08	1.21 ± 0.12	0.92 ± 0.14

Table 7.6: Performance on MolExp benchmark of different 4-agent cooperative strategies, part III.

Task	DIFF$_S$-0.001	DIFF$_S$-0.01	DIFF$_S$-0.1	DIFF$_S$-1	DIFF$_N$-0.001	DIFF$_N$-0.01	DIFF$_N$-0.1	DIFF$_N$-1
AP	0.47 ± 0.04	0.45 ± 0.05	0.45 ± 0.03	0.42 ± 0.03	0.46 ± 0.03	0.47 ± 0.02	0.45 ± 0.01	0.53 ± 0.12
A2A	0.16 ± 0.04	0.17 ± 0.04	0.14 ± 0.01	0.14 ± 0.02	0.16 ± 0.03	0.17 ± 0.04	0.19 ± 0.10	0.41 ± 0.19
BACE1	0.09 ± 0.01	0.11 ± 0.03	0.07 ± 0.02	0.06 ± 0.01	0.07 ± 0.01	0.09 ± 0.02	0.11 ± 0.03	0.10 ± 0.03
EGFR	0.12 ± 0.02	0.12 ± 0.01	0.09 ± 0.03	0.06 ± 0.02	0.10 ± 0.05	**0.16 ± 0.03**	0.11 ± 0.04	0.12 ± 0.02
Sum	0.84 ± 0.06	0.84 ± 0.07	0.74 ± 0.05	0.69 ± 0.04	0.79 ± 0.06	0.89 ± 0.06	0.86 ± 0.12	1.15 ± 0.23

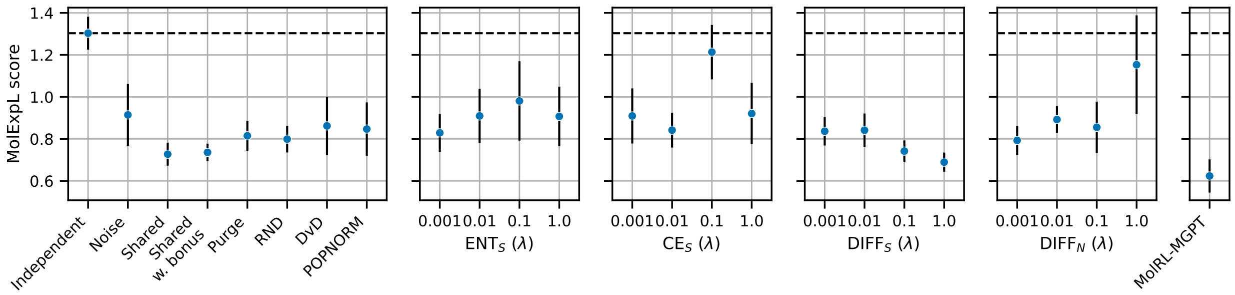

(a) Cooperative RL MolExp benchmark performance

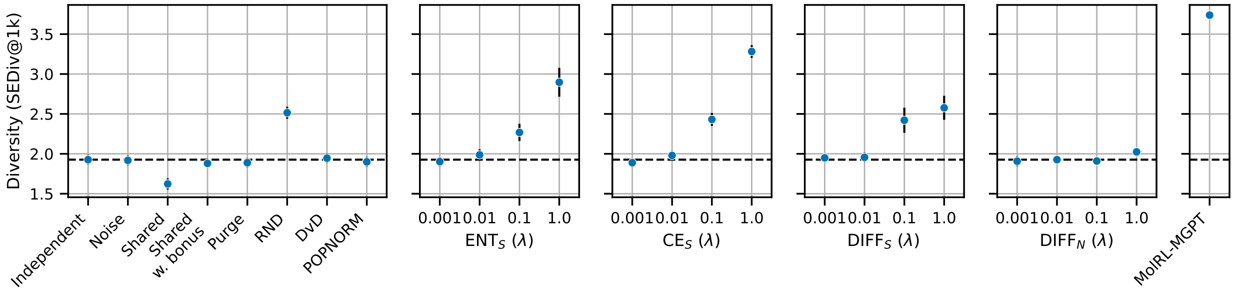

(b) Cooperative RL molecular diversity

Figure 7.4: Performance comparison of different 4-agent cooperative strategies on the MolExp benchmark, each with a budget of 10k. The dashed line represents the average of 4 independent agents as baseline.

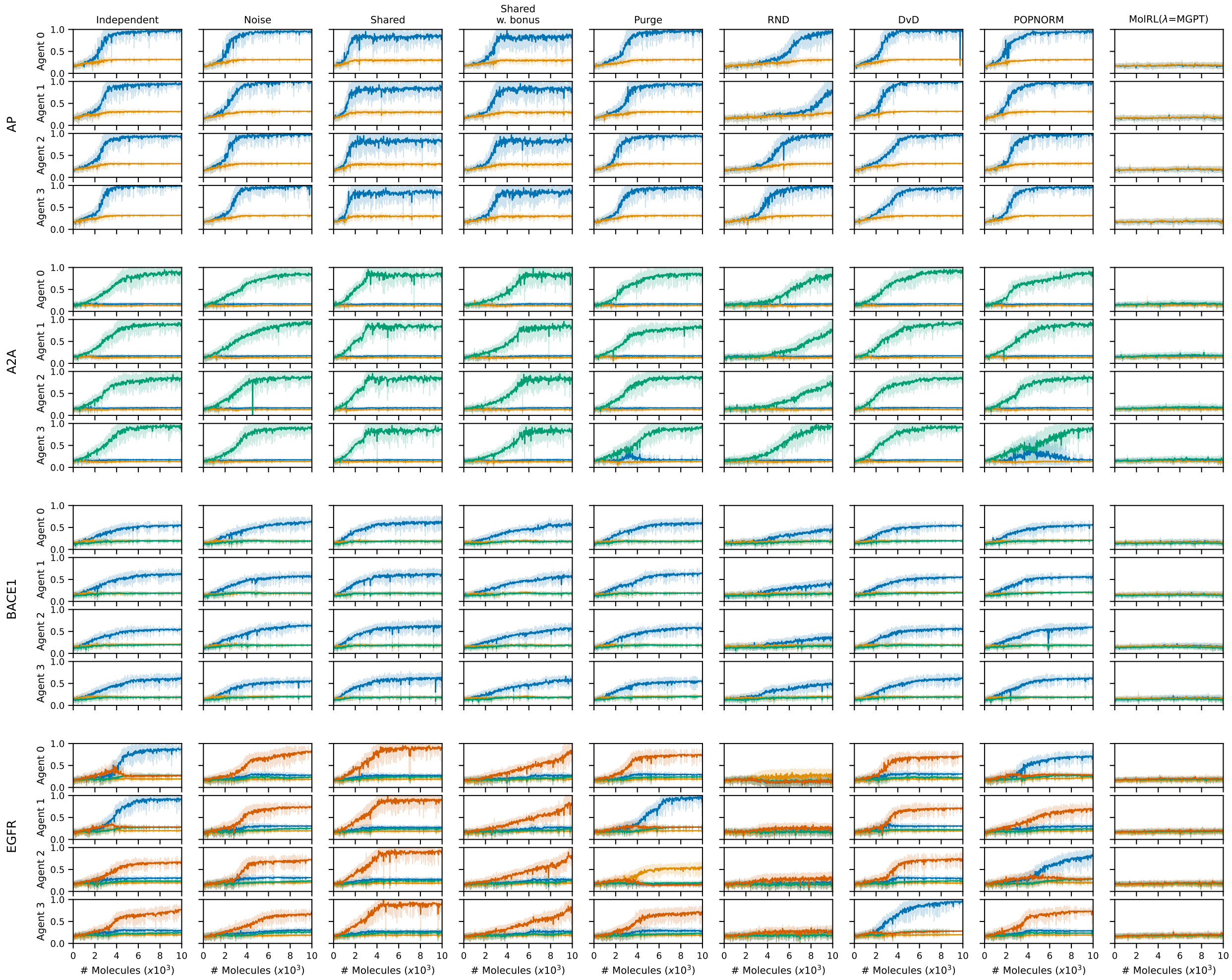

Figure 7.5: Cooperative strategies using 4-agents on the MolExp benchmark tasks, single replicate, part I. Each line represents average similarity to one of the tasks target molecules. Training of each agent is plotted. Perfect cooperation is each agent learning to rediscover a different target molecule. Most cooperative strategies result in no additional divergent behavior or in some cases, slower learning.

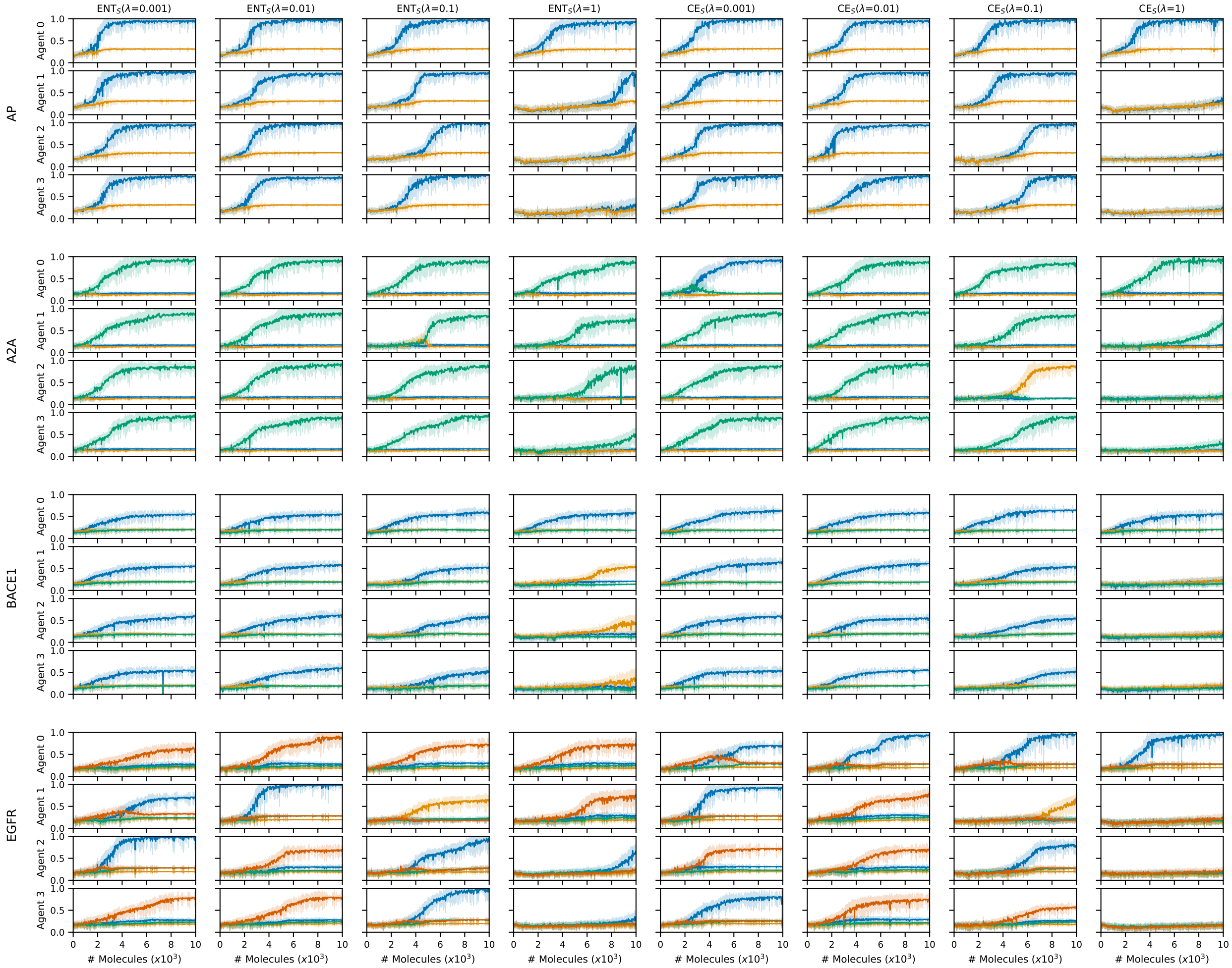

Figure 7.6: Cooperative strategies using 4-agents on the MolExp benchmark tasks, single replicate, part II. Each line represents average similarity to one of the tasks target molecules. Training of each agent is plotted. Perfect cooperation is each agent learning to rediscover a different target molecule. Most cooperative strategies result in no additional divergent behavior or in some cases, slower learning.

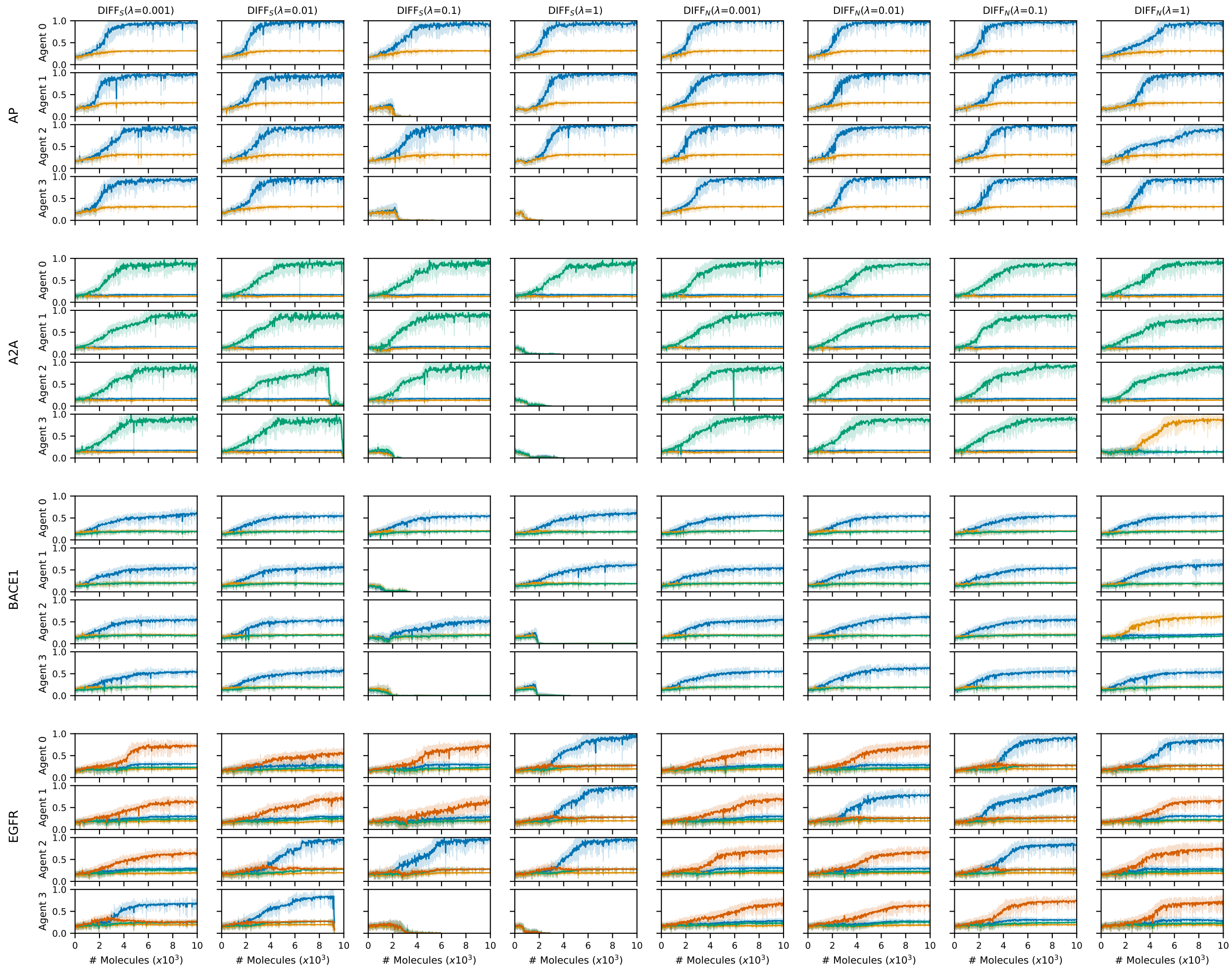

Figure 7.7: Cooperative strategies using 4-agents on the MolExp benchmark tasks, single replicate, part III. Each line represents average similarity to one of the tasks target molecules. Training of each agent is plotted. Perfect cooperation is each agent learning to rediscover a different target molecule. Most cooperative strategies result in no additional divergent behavior or in some cases, slower learning.

8 A2A bioactivity maximization

Table 8.1: Performance on MolExpBio task with increasing number of independent ACEGEN$_{MolOpt}$ agents, each with a budget of 10,000.

	1	2	4	8	16	32	64	128
Target 1 max sim	0.31 ± 0.02	0.32 ± 0.03	0.37 ± 0.03	0.36 ± 0.01	0.43 ± 0.07	0.44 ± 0.04	0.49 ± 0.07	**0.54 ± 0.07**
Target 2 max sim	0.31 ± 0.03	0.34 ± 0.03	0.36 ± 0.01	0.38 ± 0.03	0.39 ± 0.02	0.41 ± 0.02	0.43 ± 0.05	**0.44 ± 0.04**
Target 3 max sim	0.50 ± 0.08	0.53 ± 0.06	0.55 ± 0.02	0.60 ± 0.03	0.60 ± 0.03	0.62 ± 0.03	**0.63 ± 0.03**	0.63 ± 0.01
MolExpBio score	0.05 ± 0.00	0.06 ± 0.01	0.07 ± 0.01	0.08 ± 0.01	0.10 ± 0.02	0.11 ± 0.01	0.13 ± 0.02	**0.15 ± 0.02**

Table 8.2: Performance on MolExpBio task with increasing budget for a single ACEGEN$_{MolOpt}$ agent.

	10k	20k	40k	80k	160k	320k	640k	1280k
Target 1 max sim	**0.31 ± 0.02**	0.31 ± 0.02	0.31 ± 0.02	0.31 ± 0.02	0.31 ± 0.02	0.31 ± 0.02	0.31 ± 0.02	0.31 ± 0.02
Target 2 max sim	**0.31 ± 0.03**	0.31 ± 0.03	0.31 ± 0.03	0.31 ± 0.03	0.31 ± 0.03	0.31 ± 0.03	0.31 ± 0.03	0.31 ± 0.03
Target 3 max sim	**0.50 ± 0.08**	0.50 ± 0.08	0.50 ± 0.08	0.50 ± 0.08	0.50 ± 0.08	0.50 ± 0.08	0.50 ± 0.08	0.50 ± 0.08
MolExpBio score	**0.05 ± 0.00**	0.05 ± 0.00	0.05 ± 0.00	0.05 ± 0.00	0.05 ± 0.00	0.05 ± 0.00	0.05 ± 0.00	0.05 ± 0.00

Table 8.3: Performance on MolExpBio task with increasing budget for a single ACEGEN$_{MolOpt}$ agent with RND.

	10k	20k	40k	80k	160k	320k	640k	1280k
Target 1 max sim	**0.37 ± 0.07**	0.37 ± 0.07	0.37 ± 0.07	0.37 ± 0.07	0.37 ± 0.07	0.34 ± 0.02	0.37 ± 0.07	0.37 ± 0.07
Target 2 max sim	0.33 ± 0.02	0.33 ± 0.02	0.33 ± 0.02	0.33 ± 0.02	0.33 ± 0.02	**0.34 ± 0.03**	0.33 ± 0.02	0.33 ± 0.02
Target 3 max sim	0.55 ± 0.06	**0.56 ± 0.06**	0.56 ± 0.06	0.56 ± 0.06	0.56 ± 0.06	0.54 ± 0.04	0.56 ± 0.06	0.56 ± 0.06
MolExpBio score	**0.07 ± 0.02**	0.07 ± 0.02	0.07 ± 0.02	0.07 ± 0.02	0.07 ± 0.02	0.06 ± 0.01	0.07 ± 0.02	0.07 ± 0.02

Table 8.4: Performance on MolExpBio task with increasing budget for a single ACEGEN$_{MolOpt}$ agent with DF penalization.

	10k	20k	40k	80k	160k	320k	640k	1280k
Target 1 max sim	**0.31 ± 0.02**	0.31 ± 0.02	0.31 ± 0.02	0.31 ± 0.02	0.31 ± 0.02	0.31 ± 0.02	0.31 ± 0.02	0.31 ± 0.02
Target 2 max sim	0.31 ± 0.03	0.31 ± 0.03	0.31 ± 0.03	0.31 ± 0.03	0.31 ± 0.03	**0.32 ± 0.02**	0.32 ± 0.02	0.32 ± 0.02
Target 3 max sim	**0.50 ± 0.05**	0.50 ± 0.05	0.50 ± 0.05	0.50 ± 0.05	0.50 ± 0.05	0.50 ± 0.05	0.50 ± 0.05	0.50 ± 0.05
MolExpBio score	**0.05 ± 0.00**	0.05 ± 0.00	0.05 ± 0.00	0.05 ± 0.00	0.05 ± 0.00	0.05 ± 0.00	0.05 ± 0.00	0.05 ± 0.00

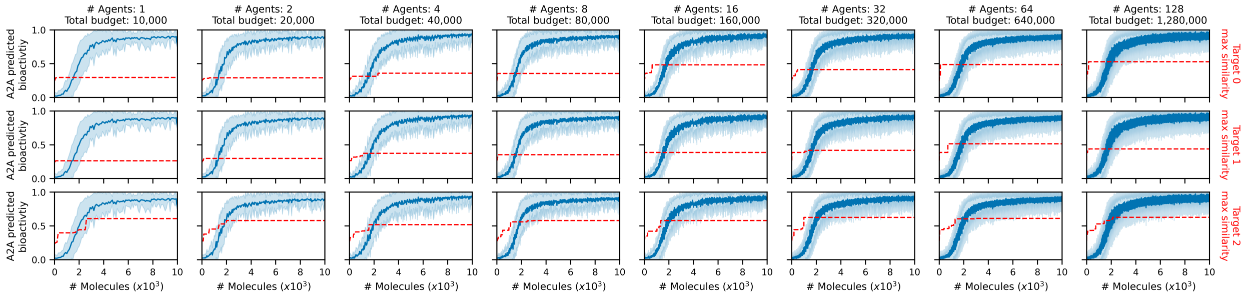

Figure 8.1: Multiple independent ACEGEN$_{MolOpt}$ agents on the MolExpBio task of maximizing A2A predicted bioactivity, single replicate. Maximization of predicted A2A bioactivity is shown, as well as the maximum similarity to each A2A target molecule in the set from the MolExp A2A task in red.

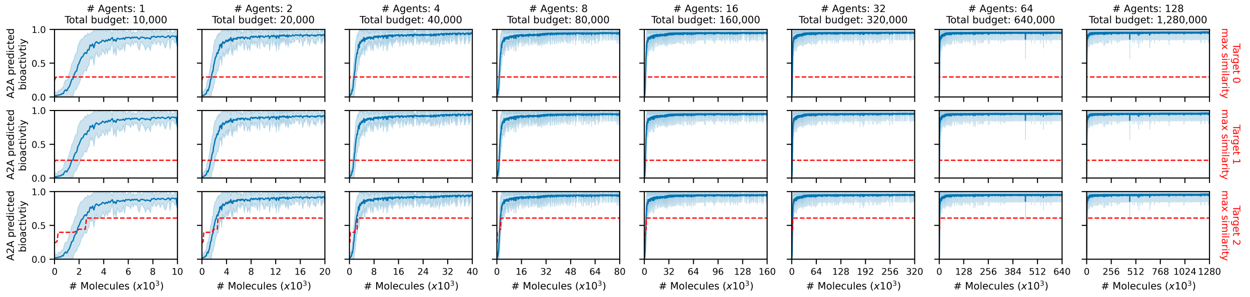

Figure 8.2: Single ACEGEN$_{MolOpt}$ agent on the MolExpBio task of maximizing A2A predicted bioactivity, single replicate. Maximization of predicted A2A bioactivity is shown, as well as the maximum similarity to each A2A target molecule in the set from the MolExp A2A task in red.

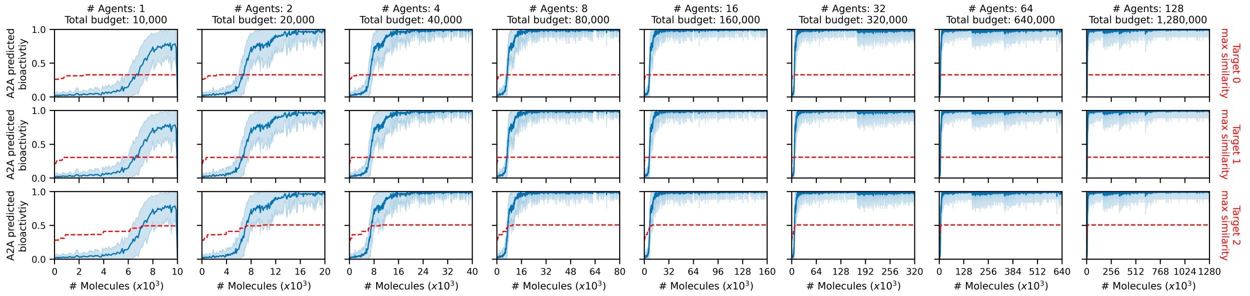

Figure 8.3: Single ACEGEN$_{MolOpt}$ agent with RND exploration bonus on the MolExpBio task of maximizing A2A predicted bioactivity, single replicate. Maximization of predicted A2A bioactivity is shown, as well as the maximum similarity to each A2A target molecule in the set from the MolExp A2A task in red.

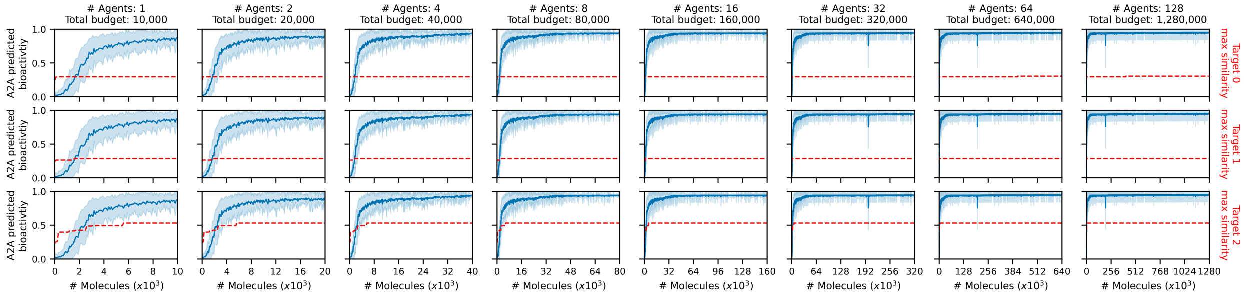

Figure 8.4: Single ACEGEN$_{MolOpt}$ agent with DF penalization on the MolExpBio task of maximizing A2A predicted bioactivity, single replicate. Maximization of predicted A2A bioactivity is shown, as well as the maximum similarity to each A2A target molecule in the set from the MolExp A2A task in red.

9 Computational resource

All experiments detailed in this work were conducted on a single consumer grade GPU, more specifically an NVIDIA RTX 3090 with 1 CPU core.

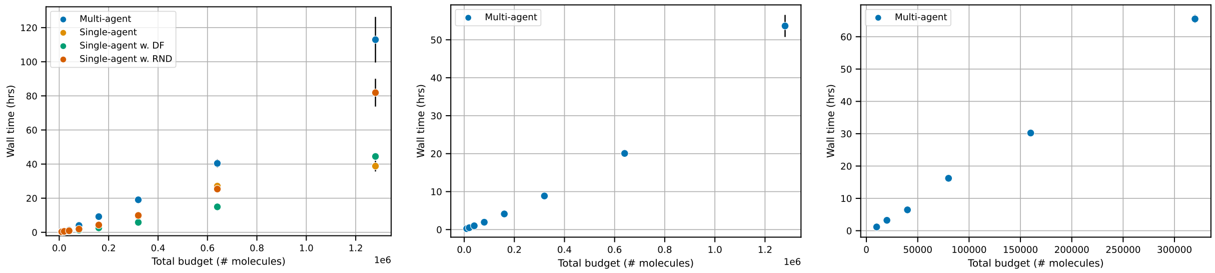

(a) MolExpL benchmark (b) MolExp benchmark (c) GuacaMol benchmark

Figure 9.1: Wall time required to run benchmarks detailed in this work. Not that the DF was implemented following some code optimizations.

References

(S1) Gaulton, A.; Bellis, L. J.; Bento, A. P.; Chambers, J.; Davies, M.; Hersey, A.; Light, Y.; McGlinchey, S.; Michalovich, D.; Al-Lazikani, B.; others ChEMBL: a large-scale bioactivity database for drug discovery. *Nucleic acids research* **2012**, *40*, D1100–D1107.

(S2) Brown, N.; Fiscato, M.; Segler, M. H.; Vaucher, A. C. GuacaMol: benchmarking models for de novo molecular design. *Journal of chemical information and modeling* **2019**, *59*, 1096–1108.

(S3) Arús-Pous, J.; Johansson, S. V.; Prykhodko, O.; Bjerrum, E. J.; Tyrchan, C.; Reymond, J.-L.; Chen, H.; Engkvist, O. Randomized SMILES strings improve the quality of molecular generative models. *Journal of cheminformatics* **2019**, *11*, 1–13.

(S4) Hu, X.; Liu, G.; Zhao, Y.; Zhang, H. De novo drug design using reinforcement learning with multiple gpt agents. *Advances in Neural Information Processing Systems* **2024**, *36*.

(S5) Olivecrona, M.; Blaschke, T.; Engkvist, O.; Chen, H. Molecular de-novo design through deep reinforcement learning. *Journal of cheminformatics* **2017**, *9*, 1–14.

(S6) Thomas, M.; O'Boyle, N. M.; Bender, A.; De Graaf, C. Augmented Hill-Climb increases reinforcement learning efficiency for language-based de novo molecule generation. *Journal of cheminformatics* **2022**, *14*, 68.

(S7) Parker-Holder, J.; Pacchiano, A.; Choromanski, K. M.; Roberts, S. J. Effective diversity in population based reinforcement learning. *Advances in Neural Information Processing Systems* **2020**, *33*, 18050–18062.
